# Study of travellers’ preferences towards travel offer categories and incentives in the journey planning context

**DOI:** 10.1371/journal.pone.0284844

**Published:** 2023-04-26

**Authors:** Eva Malichová, Milan Straka, Ľuboš Buzna, Damiano Scandolari, Mario Scrocca, Marco Comerio

**Affiliations:** 1 Faculty of Management Science and Informatics, University of Žilina, Žilina, Slovakia; 2 Cefriel, Milano, Italy; Qatar University, QATAR

## Abstract

Nowadays, efforts to encourage changes in travel behaviour towards eco-friendly and active modes of transport are intensifying. A promising solution is to increase the use of sustainable public transport modes. Currently, a significant challenge related to this solution is the implementation of journey planners that will inform travellers about available travel solutions and facilitate decision-making by using personalisation techniques. This paper provides some valuable hints to journey planner developers on how to define and prioritise the travel offer categories and incentives to meet the travellers’ expectations. The analysed data were obtained from a survey conducted in several European countries as part of the H2020 RIDE2RAIL project. The results confirm that travellers prefer to minimise travel time and stay on time. Also, incentives such as price discounts or class upgrades may play a crucial role in influencing the choices among travel solutions. By applying the regression analysis, it was found that preferences of travel offer categories and incentives are correlated with some demographic or travel-related factors. The results also show that subsets of significant factors strongly differ for particular travel offer categories and incentives, what underlines the importance of personalised recommendations in journey planners.

## 1 Introduction

Efficient public transport is one of the promising solutions for sustainable mobility [1]. Together with other sustainable modes of transportation (walking, cycling, micro-mobility options, shared transport services), it encourages multimodality and brings several positive effects such as congestion reduction, decarbonisation, physical health improvement, but also societal impacts such as increasing access to life opportunities, easing integration into society and many others [2, 3]. Although a big emphasis is currently placed on using these forms of transport, Europeans still prefer to travel mainly by private motorised vehicles [4]. It is evident that, in addition to promoting sustainable transport, it is necessary to focus on creating solutions that will facilitate people’s transition from private motorised vehicles to sustainable transport. One such solution is a multimodal journey planner.

Multimodal transport is recognised as a key element of sustainable transport, as it takes advantage of combining modes of transport [5]. Although several journey planners across Europe have been developed, many have focused only on one mode of transport or have been able to plan trips only within a particular geographical area [6]. Thus, if travellers want to plan a trip across several regions or countries, they must combine multiple journey planners, which makes trip planning difficult. Modern journey planners should provide travellers with relevant available travel solutions combining different modes of transport while considering their preferences, needs, and other factors. However, due to the consideration of multiple possible transport modes and other criteria, journey planners can overwhelm the traveller with a large number of suitable travel solutions. Hence, journey planning might be a complex decision-making situation with a plethora of influence factors and relevant criteria.

One of the ways to address this problem and provide a comprehensible and straightforward way of presenting travel solutions is the usage of the categorisation technique, often applied in recommender systems. Categorisation, in this case, is understood as assigning a specific label (e.g., cheap) to travel offers based on their characteristic properties (e.g., low price). When presented to the travellers, this travel offer label should facilitate their decision-making process, as they will be able to recognise faster travel offers that match their needs. Furthermore, categories can be used as features describing travel offers in various machine learning tasks (e.g., building a filter that will rank travel offers based on previous choices of a given or similar traveller). Incentivisation is another tool used in the recommender system that a service provider can use to affect decisions taken by travellers. It is possible to use various incentives to motivate travellers to modify their original travel decisions for some reward, whether financial or non-financial. In the context of journey planning, incentives can represent an important mechanism for changing initial travel decisions into more sustainable ones. Here, we are concerned with the question: How should we define categories and incentives and which of them could be the most suitable for these tasks? Therefore, this paper defines travel categories and incentives and focuses on analysing travellers’ preferences towards them.

## 2 Literature review

In an effort to improve the quality of sustainable transport and facilitate its usage, many initiatives are being taken across Europe. An important role among them is the development of multimodal cross-border journey planners [7–10]. Currently, unimodal, urban and regional journey planners dominate [6, 11], as the development of large-scale European multimodal journey planners is hampered by insufficient travel data integration, a large number of travel service providers, and the requirement of significant investments to maintain it.

Typical travel planner offers information on the route, distance, schedule, timing, and additional options such as schedule comparisons, fare calculations, integrated ticket purchase and information about points of interest. Nevertheless, a journey planner could foster sustainability, flexibility and resilience [11, 12]. It could combine a wide range of transport modes (public, private), be capable to react to real-time events that impact the transport network and be able to re-plan travellers’ trips automatically [13, 14]. Furthermore, it could be considered as a key to the future of seamless door-to-door travel experience by nudging traveller behaviour towards active modes through incentives and travel information [15, 16]. In order to ensure that journey planners will reach the desired impact on society, they should facilitate the traveller’s decision-making or even guide travellers to more sustainable travel solutions. Tools that can be used to achieve these goals are travel recommendations based on travellers’ preferences or previous journeys and the use of incentives.

### 2.1 Travel solutions recommendation

The task of journey planners is to recommend a travel offer (single travel connection described by attributes such as time, location, and transport mode) or travel solution (single or multiple travel offers, representing a possible realisation of a trip) that ensure travel from the origin point A to the destination point B while possibly combining several modes of transport. After presenting travel solutions to a traveller, a selection of one of the solutions should follow. Choosing a travel solution is a demanding process, and it might be impacted by many factors. To simplify this task, traditional journey planners implement filters to rank travel solutions by a criterion selected by the traveller. Consequently, this provides rather unilateral view on travel solutions and does not facilitate their comparison.

To reduce people’s cognitive effort and to reduce the need to search for additional information, the utilisation of recommendation systems is a possible solution. The recommendation systems are indispensable decision support tools in different application areas such as restaurant services [17], tourism [18], energy efficiency [19], social networks [20–22], teammate networks [23] and in numerous others. They were designed to provide meaningful recommendations based on users’ preferences and collaborative filtering of information. To take the needs of travellers into account, journey planners may consider traveller profiles and past interactions with the system [14, 24]. Such an approach should increase the diversity of choices and prevent recommending the most popular options [25]. This can be achieved by evaluating candidate journey plans, e.g., considering the price, emissions, or traveller convenience [26].

Although by using the recommender system, the journey planner can present travel solutions based on their previous trips, it is still up to the travellers to recognise whether the given solution fulfils their requirements. For this purpose, the concept of categorisation can be used, i.e., assigning the label (category) to a travel solution can help travellers efficiently sort out a large number of travel options in multimodal networks. Categories may help to choose between different travel solutions by grouping multimodal journey offers based on pre-defined factors [27]. Although there are many studies in this area, we find only a few examples addressing the categorisation of travel solutions. In 2016, Barsky and Galtzur [28] proposed the promotion of sustainable transport modes through a trip planning application, which categorised travel solutions based on departure and arrival times, price of tickets, sustainability (CO_2_ emissions), and calorie consumption. In the project SMaRTE [29], 14 categories for travelling by metro, tram, taxi, car, bus and train (e.g., cost, traffic, speed, reliability, possibility to socialise, etc.) and 13 categories for travelling by train, coach, car, plane (e.g., cost, time, flexibility, comfort, safety, accessibility, etc.) were defined to compare travel solutions. Such well-defined categories can streamline the travellers’ decision-making process and also provide an opportunity to promote selected travel solutions.

The existing research applying travel offer categories in a journey planning context can provide valuable inspirations on how to define categories, however, it does not provide a comprehensive set of categories to be implemented by a journey planner. Some useful travel offer categories can also be derived from the knowledge of factors influencing travel behaviour. Hamidi and Zhao [3] examined influence of attitudes, access possibilities and skills and competencies on mobility and travel behaviour. By applying multinomial logistic regression, they found that in addition to the price of tickets and the departure and arrival times, which are the essential factors influencing travel choices, travellers make decisions based on their attitudes, opinions, and habits. Several studies have focused on identifying these factors. Clauss and Doppe [30] summarised the factors influencing travel solution choice and grouped them into instrumental (general practical aspects of travel choice), affective (aspects linked to individual preferences), and symbolic (social expression and social identity) groups. They used a repertory grid methodology to obtain data and then aggregated it by bootstrapping to groups. Their research confirmed the impact of 28 factors on travel mode choice that can be organised into six perceptual dimensions (affection, convenience, stress, individuality, cost, and flexibility). They also analysed factors’ importance, finding privacy, autonomy, stresslessness, flexible route choice and sustainability to be the most impactful. Golightly et al. [31] considered the number of interchanges and the possibility of travel from door to door as essential factors influencing travel choices. For some travellers, minimising the distance between the exact start/end location and the start/end point of the travel (travel offers proximity) is crucial when choosing a travel solution. The importance of travel offers proximity was also confirmed by Lem [32] in the context of carpooling. Results of the multinomial logit model confirmed that the main factors influencing the choice of a travel solution are travel time, costs and distance from the carpool meeting point. The service quality attributes are also crucial for the selection of travel solutions. Hansson et al. [33], by analysing literature using PRISMA method, identified four attributes that influence modal choice and customer satisfaction: costs, comfort, punctuality and availability (frequency). Even though these studies differ in their data collection and methodology approach, they provide a set of factors that influence travel choices and, therefore, could be used to categorise travel offers.

### 2.2 Incentives

In addition to recommendations, the impact on travel decisions can also be achieved by incentives [34, 35]. There are two basic types of incentives: financial and non-financial. When choosing a travel solution, travellers focus primarily on reducing travel costs in terms of travel time or financial resources spent on travel. Therefore, financial incentives have been the most often used tools to promote sustainable modes of transport [36]. Although such a form of incentives brings a noticeable change in people’s travel behaviour [36, 37], it also undermines the intrinsic motivation to engage voluntarily, which leads to reducing interest in the promoted transport mode in the event of the removal of the incentives, especially in the case of free use or price discounts [38]. On the contrary, intrinsic motivation leads to greater engagement and better results over a longer period but needs to be backed up by extrinsic motivators to incentivise people to do tasks that do not appear inherently interesting or enjoyable to them, thus expanding its reach and efficiency [39, 40]. Therefore, the use of non-financial incentives is increasingly coming to the fore. Non-financial incentives target psychological, social, and emotional needs. These incentives enhance travellers’ self-image (e.g., by promoting their best behaviours concerning social, health or environmental matters as well as their ability to save time and money) and to avoid the negative impacts of their actions (sometimes it might be more effective to highlight the bad aspects of a given behaviour instead of emphasising the positive ones, like showing the increase of CO_2_ consumption of a specific transport solution instead of the amount a different option would save) [41]. Thus, this type of incentive is characterised by the provision of information (e.g., about travel time, calorie consumption), points for a choice of more sustainable transport modes or features such as the possibility of sharing trips with other people, e.g., through social media or use of gamification (sharing information about achievements with other travellers to spark the contest) to increase traveller’s engagement and interest [28, 42, 43].

Incentives, whether financial or non-financial, have so far been applied primarily territorially, in a certain area in which a change in users’ travel behaviour was expected and regardless of individual traveller preferences. As in the case of travel offer categories, due to the use of smartphone applications in travel, taking into account the subjective needs and preferences of travellers, i.e., personalisation, has become also demanded in the case of incentives [44–46]. This way it is possible to recommend appropriate incentives to travellers based on individual characteristics and travel preferences, e.g., to high-income travellers information about travel time and its changes and to young people who prefer entertainment while travelling various gamification activities [44]. Another example is a system that personalises the reward in the form of points to influence travellers’ decisions [45]. However, the efforts to incorporate such a system into a journey planner are limited. Mobility management tools such as Metropia or IncenTrip already contain some incentive instruments. As incentives, they use credit points, which can be exchanged for monetary rewards, transit passes, rideshare, or cash [44]. Personalisation, in this case, consists in designing a travel plan based on personal needs, preferences and previous experiences, while providing information about points allowance and expected time savings, but it does not include customised recommendations of incentives.

### 2.3 Contribution and structure of the paper

The review of existing studies shows that although the use of categorisation and incentivisation has its justification, categories of travel solutions and incentives are not commonly defined and used in journey planners. Therefore, the paper addresses this gap and delivers the following contributions:

- By summarising state of the art, we proposed candidate sets of travel offer categories and incentives, respectively.
- We designed and conducted a survey. By analysing the collected data, we identified the declared priorities of respondents towards travel offer categories and incentives.
- To gain a deeper understanding of results and to interpret them, we employed regression analyses to explore the correlations between declared priorities of respondents and demographic or travel-related factors.

Our findings provide valuable guidelines to developers when designing a journey planner employing personalisation techniques and recommender systems. The remainder of the paper is structured in the following fashion. In Section 3, we introduce the conceptualisations of initial sets of categories and incentives, respectively, together with survey design, data collection and data analysis methods. Section 4 describes the results of the data analysis. Section 5 concludes the paper by summarising the main findings and suggesting pathways for future research.

## 3 Materials and methods

The research presented in this paper is an integral part of the H2020 European project RIDE2RAIL [7]. The RIDE2RAIL falls under the fourth Innovation Programme (IP4) [47] from Shift2Rail [48], which addresses the subject of IT Solutions for attractive railway services. IP4 efforts focus on providing a journey planner application that should enable a seamless passenger experience. The RIDE2RAIL project aims to develop solutions and tools that will facilitate the efficient combination of ride-sharing and scheduled transport services such as bus and rail, extending the number of available travel modes. A ranking algorithm has been implemented to facilitate comparison and selection among multiple transport options. The algorithm considers passenger profiles and previous choices. Travel solutions are characterised by categories that describe travel offers. Categories are used as predictors by the ranker. Moreover, in the form of visually attractive icons, they are also displayed in the journey planner together with transport solutions. Another set of icons informs travellers about the availability of incentives that travel service operators offer to promote selected travel solutions. In this paper, we present the research that has been conducted to support decisions on categories and incentives to be implemented in the RIDE2RAIL project. However, the findings can be utilised in the design of any other journey planner.

### 3.1 Terminology

To clarify the terminology used in the paper and in the survey, we provide a brief description of the main terms utilised:

- A *travel offer* is a single travel connection (i.e., a product that can be purchased by the traveller) described by a set of characteristics expressed as *travel offer features* (e.g., start and end locations, start and end time, mode of transport, etc.), i.e., a set of variable-value pairs.
- A *travel solution* is constituted by single or multiple travel offers, representing a possible realisation of a *trip*. A traveller mobility request for journey planning typically results in a set of travel solutions.
- A *traveller preference* represents the subjective desirability (i.e., a quantifiable preference weight) of a specific characteristic of an offer for a traveller. The preference weight of a traveller can change considering context-awareness, i.e., under different conditions, the traveller may have different preferences. Traveller preference can be used to filter or rank the different travel solutions for a traveller.
- A *travel offer category* can be seen as a label attached to offers having particular objective characteristics. A travel offer category is computed taking into account a set of offer features.
- An *incentive* is a technique to influence the behaviour of a traveller towards a specific travel solution. The allocation of incentives can be done by evaluating rules (incentive conditions), determining the applicability of the incentive to a given travel solution for a given traveller, and the incentive mechanisms specifying the benefit proposed to the traveller if the specific travel solution is selected.

### 3.2 Methodology

To provide an advice to journey planner developers on how to implement efficient decision support to travellers when selecting a multimodal travel solution and gives to travel service providers suitable tools to incentivise travel offers, we followed the workflow composed of the steps:

Step 1: Conceptualisation of travel offer categories.
Step 2: Conceptualisation of incentives.
Step 3: Design of the questionnaire.
Step 4: Execution of the survey.
Step 5: Analysis of the survey results.

By collecting data at a European scale, the aim is to investigate traveller preferences with respect to travel offer categories and incentives that can influence the behaviour in the journey planning context. Sections 3.3 and 3.4 describe, respectively, the conceptualizations of travel offer categories and incentives adopted and validated through the performed survey. The proposed conceptualisation, reported in the Ride2Rail deliverable D2.1 [49], resulted from a detailed analysis of state of the art and an alignment with past and ongoing European research projects.

### 3.3 Conceptualisation of travel offer categories

From the analysis of the state of the art, a set of patterns were identified to provide a conceptualisation of the term travel offer category and to formulate candidate categories [27, 49]. Each travel offer category is defined considering a set of variables to be evaluated as factors to determine the membership of an offer to a given travel offer category.

First, we considered contributions discussing the different types of variables that can be used to characterise a multimodal travel offer. Integrating the models proposed by Clauss and Doppe [30] and Zhao [50], it is possible to identify the following macro-areas:

- *Instrumental*: variables related to the measurable characteristics of the travel solution (cost, time, etc.);
- *Perception*: variables related to the users’ perception while travelling (comfort, safety, etc.);
- *Symbolic*: variables related to the personal value attributed by a user to a specific travel solution (prestige, status, etc.).

Considering these types of variables, we analysed the state of the art to identify the set of actual variables that can be used as determinant factors to formulate travel offer categories [31, 33, 51]. It is important to highlight that we considered for our selection process only objective variables describing travel offers. Indeed, the process of associating offers to travel offer categories should be objective to minimise the risk of assigning a label that may be misleading because interpreted differently by different users. We introduce this distinction to clarify the difference between the process of travel offer categorisation and the filtering and ranking based on the subjective preferences of a traveller. As a relevant example, it is not possible to univocally define an *Accessible* offer category. The accessibility of a travel offer for a traveller strictly depends on her/his needs and cannot be generically assessed. This very important topic should be considered in defining a proper set of travellers’ preferences to enable filtering of the accessible travel solutions. As an additional remark, the categorisation process should not be confused with the process of recommending travel offers labelled with certain travel offer categories to specific users. Indeed, any traveller may have different subjective preferences in the selection of travel offers belonging to one or more travel offer categories.

Following the introduced conceptualisation, we mainly focused on *instrumental* variables for the selection of variables to be considered in the categorisation process since they are objective and easily measurable. We decided to take into account also *perception* variables since an objective quantification could be evaluated through feedback collected from an adequate statistical sample of users, e.g., measuring the feeling of personal safety or the level of comfort. The same does not hold for *symbolic* variables that are more subjective, which is related to problems of cognitive effects, social desirability and unstable attitudes [52]. For this reason, they cannot be considered to characterise offers for a generic user.

For each *instrumental* and *perception* variable extracted from the literature analysis, it is possible to define a low-level offer category, i.e., a class that can be associated with an offer relying on a single determinant factor. For example, the total travel time variable is the determinant factor for a low-level offer category that minimizes the said travel time identifying the quickest solution. The determinant factors extracted from the literature are: total travel time, frequency of the service, stops required, total travel distance, variability of travel time, waiting/idle times, traffic congestion likelihood, accident or breakdown likelihood, influence of weather on travel time, total cost of the trip, integrated fare, polluting emissions, charity/volunteering activities, people sharing the travel, transfers required, different means of transport, distance on foot, distance to drive, distance from start/stop location, protection from bad weather, personal safety feeling, level of privacy, overcrowding likelihood, cleanliness of vehicle, internet access, and space available.

Given the large number of variables identified, we decided to define ten macro-categories clustering the identified determinant factors. The goal of the final list of travel offer categories is not to be exhaustive, but to elicit the most common ones that can be relevant for travellers. The catalogue of travel offer categories defines the following instances:

1. **QUICK** category measures how convenient and efficient the solution is in terms of time-related issues, considering the total travel time, the frequency of service, the waiting time between legs and the number of stops required. Real-time data on traffic congestion can also be taken into account if the solution includes a segment on-road (e.g., bus/car).
2. **SHORT** category focuses on minimising the distance covered.
3. **RELIABLE** category concerns the likelihood of delays, traffic congestion, breakdowns or last-minute changes that could affect the travel time and comfort of the trip. Some solutions are inherently variable (e.g., traffic delays when crossing a city at rush hour), while other solutions might offer a small window to change the mode of transport that could cause massive idle times. Lastly, the influence of the weather on the trip is taken into account.
4. **CHEAP** category concerns the total price of a trip, the possibility of sharing part of it with others and the ease of payment, giving additional value to solutions that offer an integrated fare system and do not require the traveller to purchase different tickets from different platforms.
5. **DOOR-TO-DOOR** category covers the distance of the traveller’s start and endpoint from the beginning and destination locations of the solution provided. It is measured by the amount of walking or driving distance the traveller has to cover.
6. **COMFORTABLE** category, concerns objective factors such as weather protection, the number of transfers required, and the number of different means of transport used, it also covers a set of other elements about the quality of the trip that has to be evaluated through travellers’ feedback. This category should consider also the likelihood of overcrowded vehicles, the feeling of personal safety, the level of privacy and the cleanliness of the stations and vehicles used.
7. **SOCIAL** category concerns the identification of offers that, based on the context and means of transport used, facilitate the sharing of the trip with other passengers and the possibility to network and socialize.
8. **MULTITASKING** category concerns the extent to which the traveller can perform other tasks while travelling. These activities can regard productivity (private or work), fitness, or enjoyment. This category considers the amount of space available, as well as, whether the internet connection is provided. Lastly, the level of privacy might also influence the extent to which a person can work and could be considered as a determinant factor for this category.
9. **ENVIRONMENTALLY FRIENDLY** category covers the green aspects of the trip, taking into account at least the amount of CO_2_ emissions measured per kilometre/traveller for each mean of transport included in the offer and considering the distance covered and the number of passengers. If available, additional determinant factors can be considered as energy consumption, NOx emissions (nitrogen oxides) and the carbon footprint.
10. **PHILANTROPIC** category relates to the willingness of the traveller to choose a solution that contributes to social causes or charity activities (e.g., donations included in the offer price).

### 3.4 Conceptualisation of incentive categories

The analysis of the state of the art discussed in Section 2.2 resulted in the identification of the following patterns to be considered for the conceptualisation of incentives that could influence the behaviour of a traveller:

- Extrinsic motivators;
- Increase of awareness on specific choices;
- Gamification strategies;
- Personalized incentives tailored to the specific user.

The identified patterns were used to identify a catalogue of candidate incentives that could be used to analyse the potential impact of different strategies on travellers. The incentives are divided between: (i) *tangible* incentives, i.e., providing a practical benefit to the traveller such as a gift or a discount (incentives 1—6), and (ii) *intangible* incentives, i.e., not employing practical benefits (incentives 7—10). The list of incentives is composed as follows:

1. **IMMEDIATE DISCOUNT**—immediate price discount on a given travel offer,
2. **FUTURE DISCOUNT**—discounts on the following purchases if a given travel offer is chosen,
3. **LOYALTY PROGRAM**—earning points associated with travel offers, while collected points can be converted to prizes,
4. **DISCOUNT FOR SERVICES**—ancillary services for free or discounted (e.g., meal),
5. **ADDITIONAL SERVICES**—discounts on complementary services (e.g,. hotel),
6. **CLASS UPGRADE**—discounted or free upgrade of the travel class.
7. **ENVIRONMENTAL INFORMATION**—provide to the traveller information that can increase her/his awareness of the environmental sustainability of a travel offer (e.g., displaying the CO_2_ emissions),
8. **ADDITIONAL INFORMATION**—provide to the traveller additional material promoting the offer, e.g., include in an offer involving a bus the images of city monuments that can be spotted during the travel,
9. **GOAL**—adopt a gamification strategy assigning badges to award the achievement of pre-defined goals (e.g., trying a ride-sharing solution for the first time, or choosing the solution with the lowest environmental impact),
10. **COMPETITION**—assign points to the travellers for virtuous choices in travel offers and set up a daily/weekly/monthly shared leaderboard (e.g., among friends).

### 3.5 Survey design

Considering the defined conceptualisation, the survey would like to validate the proposed catalogue of travel offer categories and the preference model to obtain insights on what choice criteria are more relevant for the traveller. Moreover, we would like to assess the completeness of the identified catalogues by asking the traveller to propose additional entries. This second aspect can provide valuable information also to understand if the proposed definition of the concept has been understood by the traveller. Considering incentives, similarly, we would like to investigate, through a set of examples, which of the approaches that emerged from state of the art could be more attractive for a traveller. Moreover, we would like to obtain additional suggestions on incentives that can influence the behaviour of a traveller.

The survey focused on gathering information on a choice of travel scenario considering a traveller’s perspective with their specific mobility needs. Since the choice criteria and incentives influencing the traveller behaviour depend on the specific traveller but also on the specific context describing the type of trip to be performed, we designed the initial part of the survey to let the traveller focus on a specific trip. Since the travel contexts can vary, we decided not to propose predefined contexts to choose from. Instead, we decided to ask the traveller to focus on their last trip and then describe it considering a list of context dimensions. All questions Q1-Q18 in the questionnaire are presented in Section Appendix C in S1 File.

The defined set of travel context dimensions and potential values to let the traveller describe their last trip were “reason of the trip” (Q1), “accompanying persons” (Q2), “length of trip” (Q3), “trip origin” (Q4), “trip destination” (Q5), and “means of transport used” (Q6). The central part of the survey has been designed to obtain useful insights to validate and finalise the conceptualisation of choice criteria and incentives. To do this, the travellers were asked to imagine using a travel app to plan/optimise a trip similar to the one described at the beginning by comparing different journey solutions. With this journey in mind, travellers were asked information on choice criteria (Q7-Q11) and incentives (Q12 and Q13) with a set of questions following the usual interaction order in a typical journey planning application: definition of preferences with reference to travel solutions (traveller preferences), visualisation of travel solutions (travel offer categories) and proposal of incentives for selecting different travel solutions or additional services (incentives).

We decided to place focus on the most common variables through which a traveller can set some travel preferences in Q7. We aimed to provide the respondent with a general set of characteristics applicable to different types of trips and travellers to obtain comparable answers. We selected the following: “transportation company”, “time interval for the departure and arrival times”, “number of transport changes”, “travel class”, “seat type”, “meal inclusion”, “refundability”, “live notifications on trip status updates”, and “on-board connectivity”. To investigate also additional and more specific offer features on which a traveller may be interested in expressing preferences we decided to adopt a different strategy, starting from the traveller needs. We first provided the respondents with a set of potential additional needs to choose from, and we then asked to specify, through an open-ended question, which traveller preferences related to the indicated needs that they would like to specify. The additional needs considered in Q8 have been: “large/multiple baggage/s”, “special baggage (sports equipment, instruments, etc.)”, “animal allowance”, “help needed because of reduced mobility”, “health-related needs”, “travel with an infant”, “other needs”. To investigate travel offer categories, in Q9 we asked travellers to indicate which categories they consider more relevant to discriminate among different travel solutions. This was achieved by a set of questions collecting a 1 to 5 relevance score related to each of the ten proposed travel offer categories, namely: “quick”, “short”, “reliable”, “cheap”, “door-to-door”, “social”, “multitasking”, “environmentally-friendly”, “philanthropic”, “comfortable”. The last block of journey-related questions addressed incentives. We identified different approaches in the state-of-the-art 3.4, and we proposed a distinction between tangible and intangible incentives. We included question Q12 for each one of the tangible and intangible incentives. More in detail, the tangible incentives tackled were described as: “immediate price discount”, “price discount on future purchases”, “loyalty program with points collection to unlock different rewards”, “being offered additional services”, “discounts on complementary services (e.g., hotel, restaurants, etc.)”, “free (or discounted) class upgrade”. Concerning the intangible incentives, the questions addressed the following items: “provide more information about the positive aspects of a solution”, “provide information on the solution’s environmental impact”, “challenge you to achieve a specific goal”, “competition with friends and a shared leader-board with points assigned based on your travel choices”.

To conclude the design of the survey, we selected a set of socio-demographic dimensions to be asked about in Q14-Q18. This set of variables allowed us to identify the characteristics of the population answering the survey and to check if the sample was well distributed or unbalanced towards specific values. The socio-demographic dimensions selected were: “age”, “gender”, “country of residence”, “education level” and “employment status”.

### 3.6 Data collection

The survey was implemented and administered via Coney [53], an innovative toolkit designed and developed by Cefriel to administer surveys. Coney uses a conversational approach, disguising a quantitative data collection process as a qualitative interview by administering the survey in a chat-like interface that resembles an actual conversation with the goal of enhancing the traveller experience and engagement. The toolkit offers different web applications that cover all the stages of survey design and delivery processes, from the survey creation to its administration and the subsequent data analysis.

Once the survey was implemented, both the chat interface and the survey content were translated in twelve different languages, namely: English, Italian, Greek, Finnish, Slovak, Czech, Spanish, French, German, Ukrainian, Portuguese, and Croatian. Once finalised, the questionnaire was administered via the Coney Chat web application, offering the respondents an easy-to-use chat-like interface to fill up the survey. Coney’s live dashboard, Coney Inspect, was used to keep track of the completion progress. The data collection process started on the 2nd of July 2020 and lasted around two months. The request to fill in the survey was distributed by partners of the Ride2Rail project and shared through several dissemination channels like mailing lists, social media, or websites together with an URL that opened the chat application and started the survey. The identity of survey respondents was kept anonymous. The data collection process was finalised on the 7th of September 2020.

### 3.7 Dataset

Once the data collection process ended, the gathered data was exported in CSV format and analysed. While more than 787 participants started the survey, the total number of respondents that completed the survey is 609, signalling a drop-out rate of around 22%. The average time taken to complete the survey is 8 minutes and 58 seconds and all the twelve available languages were used. To obtain a consistent sample of data a pre-processing procedure was applied. First, the data was cleaned by filtering out questionable and incomplete data (e.g., data collected during the survey preview or questionnaires with less than 80% of provided replies). We selected only the countries from which more than 80 respondents participated in the survey, while in those that were not included it was less than 30. For the analysis only the respondents’ data from Slovakia, Czechia, Italy, Finland, and Greece were used. These countries are involved in the pilots developed by the Ride2Rail project, and thus the dissemination of the information about the survey was there the most intensive. This way we obtained a dataset with 502 observations each corresponding to a respondent.

**Fig 1:**
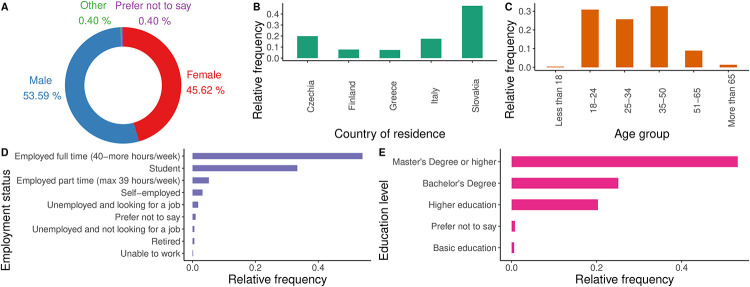
Socio-demographic characteristics of survey participants. **A** Gender distribution. **B** Country of residence. **C** Age distribution. **D** Employment status. **E** Education level.

The survey featured several socio-demographic questions examined in Fig 1. The participants included in the analysed sample mostly identify as males or females, with a good balance between the two (53.59% males and 45.62% females). Most of the respondents were between 18 and 50 years old, with a good representation recorded for the 51–65 group and very few answers collected from people below 18 or older than 65. While most of the respondents were full-time workers (54.01%), a significant amount of students (33.33%) were also recorded. With regards to education level, almost all the participants achieved at least a higher education diploma, with the majority of participants having obtained a Master’s Degree or more. Only three persons selected the basic education and so we merged this category with higher education to the category without university degree. Further, we merged five occupation categories (Unemployed and looking for a job, Unemployed and not looking for a job, Unable to work, Prefer not to say) into one category Other, as they had only 23 occurrences altogether. Similarly, three categories Employed full time, Employed part time and Self-employed were merged into the Employed category. The representation of the countries in the sample is not balanced but given that the goal is to create one system for all countries participating in the demonstrations and propose one list of travel offer categories and incentives, it is not considered crucial. Nevertheless, considering the total population of these countries, with a 5% margin of error, a 95% confidence interval, the sample can be considered representative [54].

### 3.8 Data representation

To apply the regression analysis, the data was described by variables introduced in Appendices A and B in S1 File. The variables are identified by variable names that are further used in the presentation of results of the regression analysis. The provided description indicates the meaning of variables and the way how they encode the data. Table in Appendix A in S1 File presents the response (dependent) variables, each corresponding to either one travel offer category or incentive type. The preferences of survey respondents expressed on the 5-star scale are represented by ordinal variables taking integer values ranging from 1 to 5. In Appendix B in S1 File we present the explanatory (independent) variables that describe the answers of respondents to questions related to the last trip, traveller preferences and their basic socio-demographic characteristics.

### 3.9 Ordinal response regression

The 5-star rating was used in the survey to quantify the level of preference towards travel offer categories and incentives and thus it is necessary to use a method that is able to handle ordinary response variables. In addition, we wanted to provide a picture of the basic data dependencies and identify the most vital factors influencing travellers’ offer and incentive choices. Therefore, we apply ordinal response regression [55], commonly used in research related to travel behaviour [56–59], to model the dependency of ratings on the participants’ current travel behaviour, travel preferences and socio-demographic characteristics. The response variable *Y* represents the number of stars assigned by participants to a given offer category. The symbol *π*_*j*_ denotes the probability that *Y* takes the value *j* for *j* = 1, …, 5, i.e., *π*_*j*_ = *P*(*Y* = *j*). Hence, the cumulative probability for the assigned number of stars *j* of *Y* is *P*(*Y* ≤ *j*) = *π*_1_+ …+ *π*_*j*_ for *j* = 1, …, 5. The regression model examines the effects of explanatory variables *x*_1_, …, *x*_*p*_ on the cumulative logits,

$$\begin{array}{c} \text{logit}\left(P\left(Y\leq j\right)\right)=\text{log}(\frac{P(Y\leq j)}{1-P(Y\leq j)}). \end{array}$$ (1)

The model assumes that the logit of cumulative probabilities changes linearly with the explanatory variables, *x*_1_, …, *x*_*p*_, i.e.,

$$\begin{array}{c} \text{logit}\left(P\left(Y\leq j\right)\right)=\beta_{j0}+\beta_{1}x_{1}+\ldots +\beta_{p}x_{p}\;\;\text{for}\;j=1,\ldots ,4. \end{array}$$ (2)

Consequently, the model for the cumulative probability takes the form

$$\begin{array}{c} P\left(Y\leq j\right)=\frac{\text{exp}(\beta_{j0}+\beta_{1}x_{1}+\ldots +\beta_{p}x_{p})}{1+\text{exp}(\beta_{j0}+\beta_{1}x_{1}+\ldots +\beta_{p}x_{p})}\;\;\text{for}\;j=1,\ldots ,4, \end{array}$$ (3)

where *P*(*Y* ≤ 0) = 0 and *P*(*Y* ≤ 5) = 1. Values of regression coefficients were found by using the porl() function from the MAAS library available in the CRAN repository of R language. The significance of regression coefficients is evaluated based on p-values that were calculated from t-values provided by the porl() function. The p-value is calculated by considering the normal distribution *N*(0, 1) as probability of observing a value that is more distant from zero than the t-value. Hence, small probabilities indicate that the t-value is reliably distinguishable from zero. If a calculated p-value is less than 0.05, we consider the corresponding regression coefficient as significant. For the presentation purposes, in Figs 5 and 6 we report values of exp(*β*_*i*_) for *i* = 1, …, *p*, hence, value larger (smaller) than 1 (0) indicates positive (negative) impact of an explanatory on a response variable.

## 4 Results

First, we present the exploratory analysis of ratings assigned by respondents to individual travel offer categories and incentives. Second, we present the findings regarding the relationship between ratings and explanatory variables obtained by the ordinal response regression.

### 4.1 Exploratory data analyses

**Fig 2:**
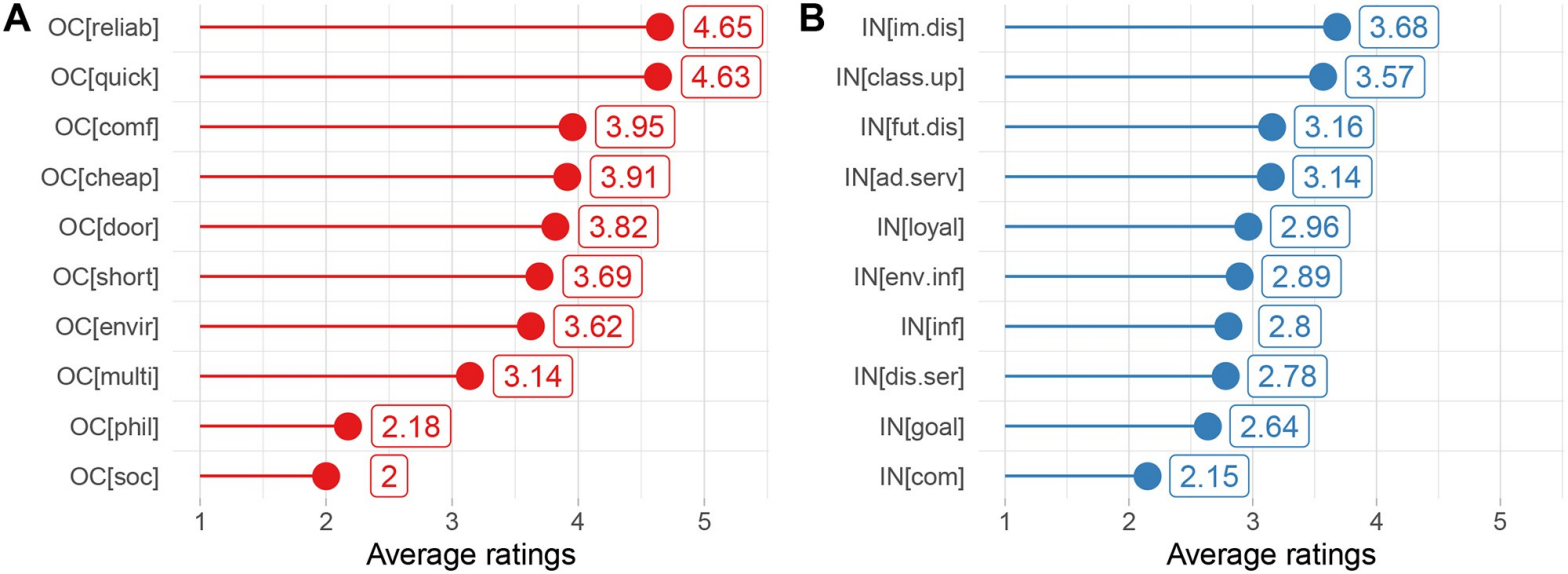
**A** Average ratings of travel offer categories. **B** Average ratings of incentives.

**Fig 3:**
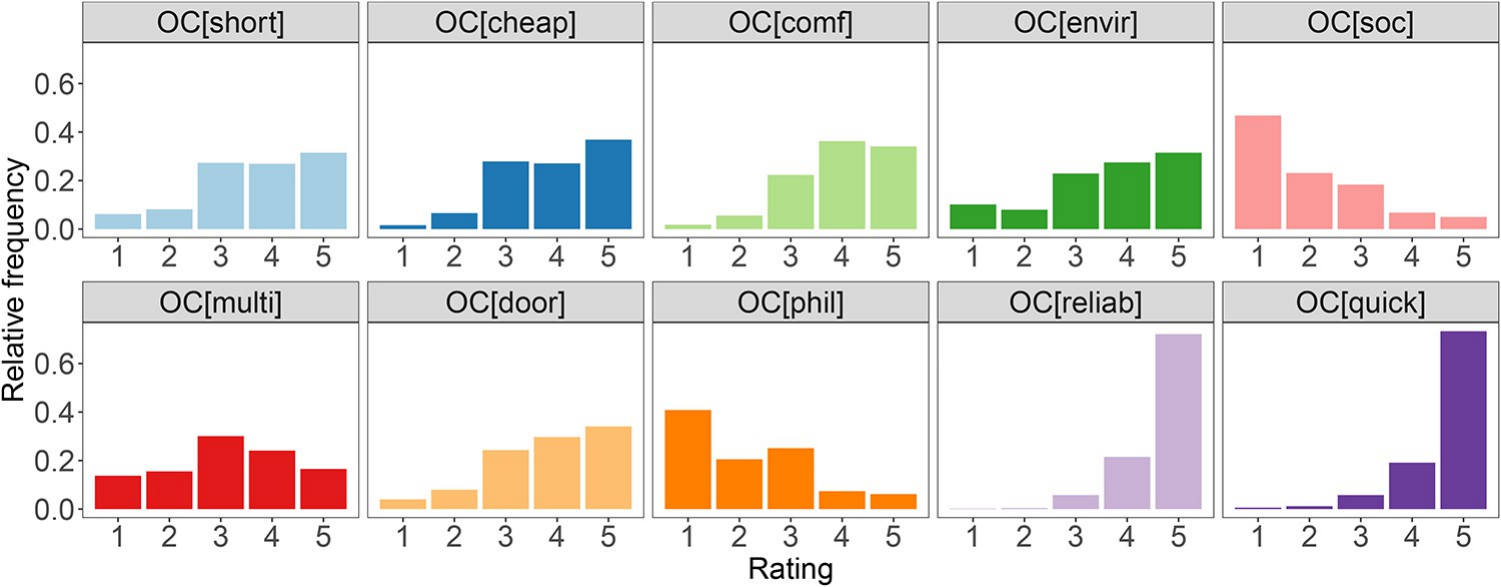
Travel offer categories ratings distributions.

The first impression about perceptions of travellers regarding travel offer categories provides the average ratings presented in Fig 2A. The ratings go from 1 (not important) to 5 (very important), and they were provided as a reply to question Q9. On average, most of the travel offer categories were ranked higher than 3 out of 5, hence in the upper part of the range. The “reliable” and “quick” were highly preferred among the respondents. More than 60% indicated these categories as the most relevant. The distribution of ratings, presented in Fig 3 confirms that only a few respondents assigned to these categories rates 1 and 2. It means that most people would like to be informed whether a travel solution is among those that demand as little time as possible or those where the probability of a change in travel time due to delays, traffic jams or breakdowns is not high. The second group of categories, “comfortable”, “cheap”, “door-to-door”, “short”, “environmentally friendly” and “multitasking” reached average ratings between 3 and 4 and featured very similar rating distributions. The lowest interest was found in the case of “philanthropic” and “social” travel offer categories, where the majority of respondents assigned the rating 1.

After rating travel offer categories, respondents were asked to select the three most important ones. The purpose was to validate and identify priorities since previously the ratings were collected individually. The top five most frequently selected travel offer categories match those presented in Fig 2. The category “quick” was selected by 81.1%, “reliable” by 74.5%, “cheap” by 46.7%, comfortable by 27.4% and door-to-door by 24.9% of respondents.

**Fig 4:**
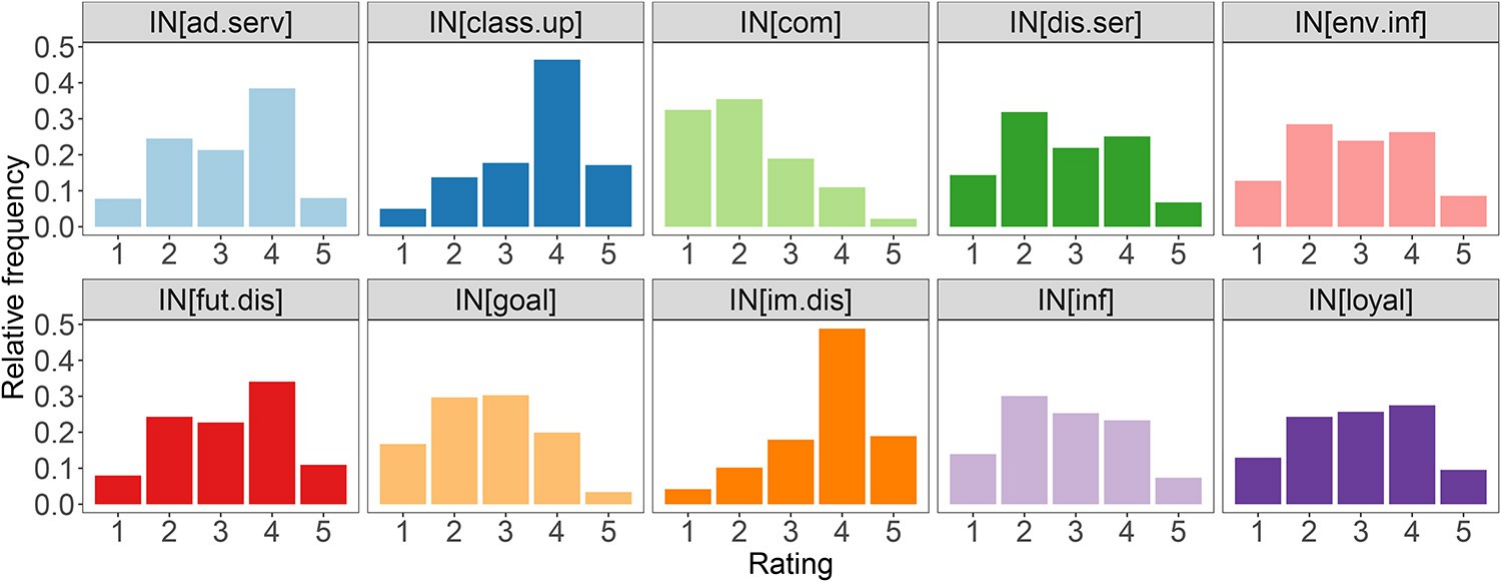
Incentives ratings distributions.

We applied a similar approach to the incentives by asking the respondents in question Q11 to rate them based on how effective they could be. The average ratings are presented in Fig 2B. The average ratings are closer to the value of 3, which is the midpoint of the rating interval. The respondents would be willing to change their travel choices mainly because of “immediate price discount” and “discounted class upgrades”. Thus, the money-related tangible incentives are among the highest-rated. The distributions of ratings for these two incentive types (see Fig 4) are very similar, reaching the maximum at the value of 4. The average value close to 3 we find for “price discounts on future purchases”, “discounts on complementary services”, “loyalty program”, “information on travel solution’s environmental impact”, “information about positive aspects of a travel solution”, and “discount on complementary services”. All these incentives have relatively uniform rating distributions with a slight preference towards the mid values. On the contrary, the incentives that are the most unlikely to change the respondents’ choices are the “challenge of achieving a specific goal” and “competition with friends”, which are intangible incentives.

Respondents were also asked to list any additional incentives that could influence their choice of the means of transport. Several of them stated that the main incentive would be to get further discounts, especially those targeting specific groups like students, youngsters, or older people. Another suggested incentive is to offer travel insurance (covering health issues during the trip or delays and cancellations) included in the ticket price. Several respondents mentioned a free cancellation policy or a possibility to change the ticket booking freely, free food during the trip, food with gluten-free or vegan choices, sharing of the information or the proposition of other activities (e.g., a possibility to visit a museum or a possibility to take some historic or newly adopted means of transport).

### 4.2 Results of the ordinal response regression

The purpose of the categorical regression analysis is to tackle the following questions:

1. Who and in what context is interested in presenting travel offers together with examined categories and incentives?
2. How is it possible to tailor the presentation of travel offer categories and incentives to a specific group of travellers?

Travel offer categories are in the Ride2Rail project used together with other traveller and trip-related information as features to build a prediction model of purchase decisions. Such a model can be used to rank the offers, and this way simplifies the selection of the travel offer for a traveller. Hence, the analyses could give us insights on quantities represented by categories in the prediction of purchase decisions. Except for travel offer categories quick and reliable, we created ordinal regression models for each offer category and incentive. Models for these two categories could not be created because the distribution of respondents’ answers (see Fig 3) means that the choice of these categories is not dependent on specific factors, but it is a general interest of all participants to prefer travel and reliable travel options.

Tables 1 and 2 show McFadden’s and Nagelkerke’s *R*^2^ of the created models. In general, realistic values of the proportion of variability in response variables explained by explanatory variables might be in domains like psychology and marketing well below 0.1 [60]. Although the achieved values of McFadden’s *R*^2^ are relatively low (from 0.045 to 0.097) it must also be taken into account that explanatory and response variables are categorical, for which *R*^2^ type of measures are typically lower than for ordinary least squares. The author of the McFadden’s *R*^2^ coefficient stated that values between 0.2 and 0.4 of this coefficient represent an excellent fit [61]. To provide additional information about the fit, we evaluated the Nagelkerke’s *R*^2^ [59], which extends commonly used Cox-Snell *R*^2^ [58], by scaling it between 0 and 1.Nagelkerke’s *R*^2^ reaches higher values than McFadden’s *R*^2^ and it does not fall below 0.1. As both coefficients are computed from the model likelihood, their values are correlated. Models having best fit are OC[cheap], OC[comf], OC[phil], IN[class.up], IN[ad.serv]. To check the overall significance of models, i.e., if the coefficients are different from zero, we performed the likelihood ratio test by ANOVA [55]. All the models had p-value below 0.05 and hence we consider them as significant.

**Table 1:** McFadden’s *R*^2^ for travel offer categories models.

category	OC[short]	OC[cheap]	OC[comf]	OC[envir]
McFadden’s *R*^2^	0.054	0.093	0.097	0.064
Nagelkerke’s *R*^2^	0.153	0.234	0.241	0.182
category	OC[soc]	OC[multi]	OC[door]	OC[phil]
McFadden’s *R*^2^	0.080	0.052	0.069	0.089
Nagelkerke’s *R*^2^	0.207	0.157	0.186	0.235

**Table 2:** McFadden’s *R*^2^ for incentive categories models.

category	IN[inf]	IN[env.inf]	IN[goal]	IN[loyal]	IN[fut.dis]
McFadden’s *R*^2^	0.052	0.059	0.064	0.045	0.046
Nagelkerke’s *R*^2^	0.154	0.172	0.179	0.134	0.136
category	IN[im.dis]	IN[dis.ser]	IN[class.up]	IN[ad.serv]	IN[comp]
McFadden’s *R*^2^	0.066	0.049	0.082	0.083	0.057
Nagelkerke’s *R*^2^	0.173	0.144	0.217	0.227	0.155

**Fig 5:**
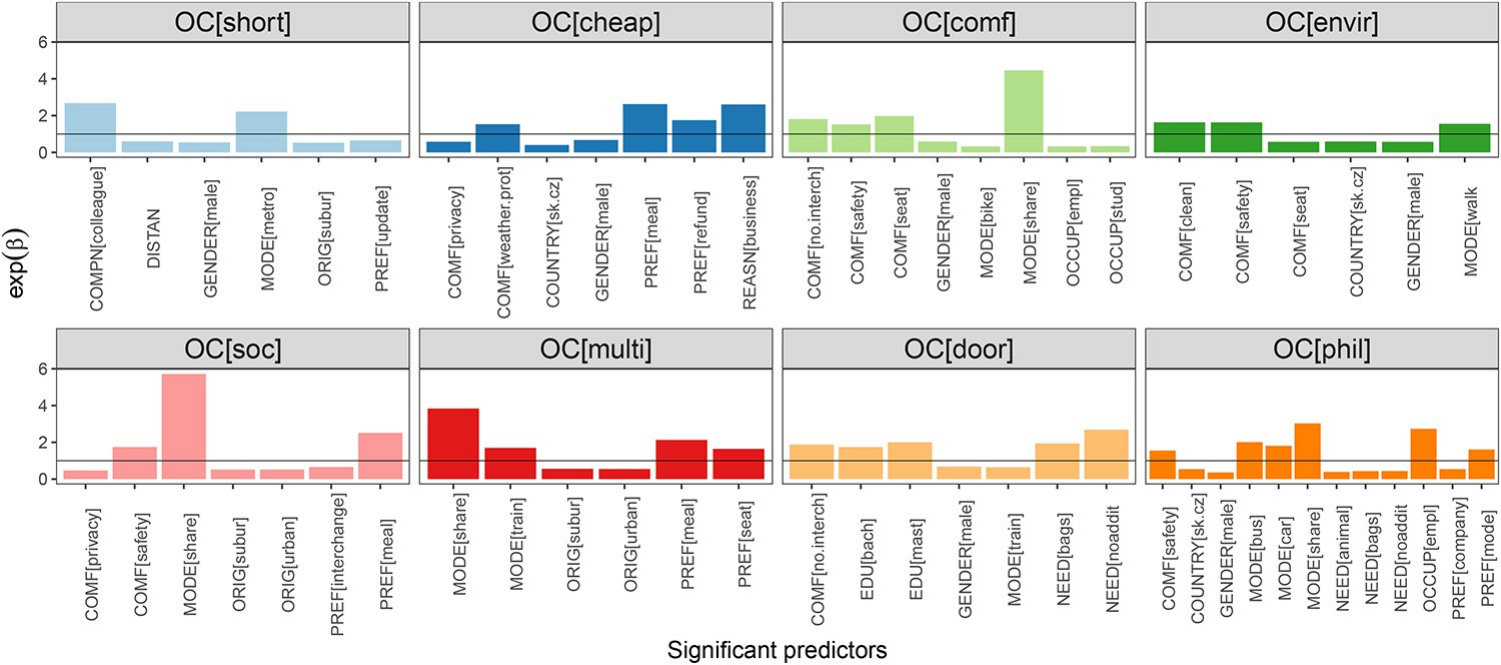
Values of regression coefficients obtained by applying ordinal response regression to travel offer categories. Only values of exp(*β*) corresponding to statistically significant predictors are shown. By the horizontal line we indicated the value 1.0, which is the borderline between predictors with a positive and negative impact on the response variable.

**Fig 6:**
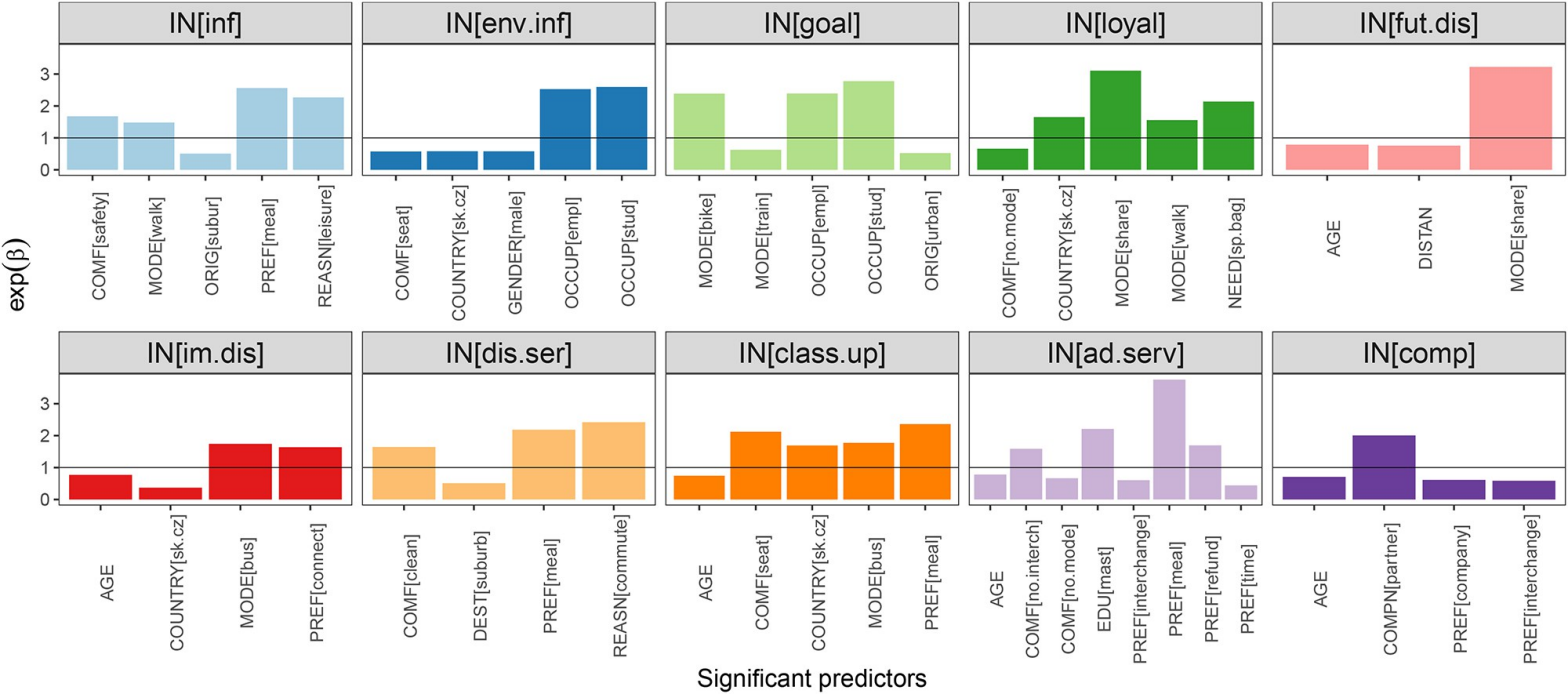
Values of regression coefficients obtained by applying ordinal response regression to travel offer categories. Only values of exp(*β*) corresponding to statistically significant predictors are shown. By the horizontal line we indicated the value 1.0, which is the borderline between predictors with a positive and negative impact on the response variable.

The statistically significant explanatory variables with values of exp(*β*) resulting from these models are presented in Figs 5 and 6. Values of regression coefficients together with p-values are presented in Appendices D and E in S1 File. Since regression analysis results strongly depend on categories and incentives, we describe separately the findings for each travel offer category and incentive. Both are sorted based on their ratings resulting from the exploratory data analyses. In addition, we also focus on the implications of our findings for the possible utilisation of travel offer categories and incentives in recommender systems.

#### 4.2.1 Results: Travel offer categories

Among those travel offer categories for which a model has been created, the most relevant is “comfortable”. The results show that this category could be interesting for travellers who used ride-sharing, carpooling or shared taxi services on their last trip. Other factors that positively affect the choice of this category are factors defining a comfortable solution: “a minimum number of interchanges”, “feeling of personal safety”, and “having a comfortable seat”. These comfort factors could be important elements when developing an algorithm to recommend a travel offer because they are clearly linked to the “comfortable” travel offer category, and at the same time, they are consistent with the identified significant explanatory variable: transport mode “carpooling/ride-sharing/shared taxi”.

The significant factors positively influencing interest in the “cheap” travel offer category are the “reason of a trip (business)”, traveller preferences “meal inclusion” and “refundability”, and comfort factor “weather protection”. Contrariwise, males, people from Czechia and Slovakia, and those who prefer privacy during travel are less interested in inexpensive travel options. It is likely that people who desire to seclude themselves from other passengers are willing to pay extra and therefore do not seek cheap travel solutions. We also found that participants from Czechia and Slovakia where the average monthly income is less than in other analysed countries are not interested in spending less money on travelling. There are several possible reasons which contribute to this situation, e.g., different transport costs across countries, price discounts for some specific groups, quality of the service, culture, etc. Identified influential preferences are closely related to the reason of the trip. People travelling for work-related purposes often prefer to eat while travelling or have a possibility to refund costs in case of cancellation.

As expected, people who prefer a minimum number of interchanges during their journeys are interested in the “door-to-door” travel offer category. The results also show that this category is relevant for people typically travelling with large/multiple baggages or who do not have additional needs. Interestingly, people with a university degree are more likely to be interested in the “door-to-door” category.

It can be seen that the “short” travel offer category tends to be preferred by people whose last trip was performed by metro, and they did not travel alone but with colleagues. The interest in this offer category decreases when travellers are men, travel from a suburban area with respect to the reference category (rural area), or the distance of a trip is longer. Also, people who want to receive updates about trip status are less interested in “short” travel options, as they are probably more interested in other factors than distance (e.g., travel time). These results suggest that people travelling over shorter distances try to minimise them even more, especially within the city using fast public transport.

Naturally, people who walked on their last trip are more likely to be interested in the “environmentally friendly” travel offer category. In addition, a significant positive impact was also identified for people who considered personal safety and cleanliness of stations and vehicles as a comfortable solution. Thus, if implemented into a recommender system, this travel offer category, is likely to be appreciated by travellers interested in active modes of transport and travellers sensitive to the safety and cleanliness of public spaces.

Interest in the “multitasking” travel offer category depends on a transport mode, traveller preferences and origin of a trip. People who travel by shared services (carpooling/ride-sharing/shared taxi) or train are more willing to appreciate this category than those travelling by other transport modes. This also applies to people whose preferences are “meal inclusion” and “seat type”. In addition, people travelling from urban and suburban areas are less interested in multitasking travel solutions than people travelling from rural zones. Hence, people who typically travel from rural to urban areas might be more used to working, studying or doing other activities during their trips because of longer travel time and therefore be more interested in the multitasking category.

The least interest of respondents was identified for the “social” and “philanthropic” travel offer categories. In “social” category are interested people who used a shared service (carpooling/ride-sharing/shared taxi), people who defined as a comfortable factor “feeling of personal safety” and people whose preference is “meal inclusion”. Since 12 significant explanatory variables have been identified for the “philanthropic” travel offer category, it is difficult to derive a recommendation regarding an interested group of travellers.

#### 4.2.2 Results: Incentives

Respondents indicated that they would be willing to change their travel choices mainly due to financial incentives. The results of the ordinal regression in Fig 6 show that the respondents who travelled by bus on their last trips and respondents who selected onboard connectivity as travel preference are more likely to choose “immediate price discount” to change their travel choice. Interestingly, respondents from Czechia and Slovakia are less interested in price discounts than respondents from other countries. This finding is well aligned with the findings about the “cheap” travel offer category. On the contrary, these respondents are more likely to select the “free(or discounted) class upgrade” incentive. In addition, a significant positive impact on this incentive was also identified for a bus as a transport mode, comfort factor seat availability and travel preference meal inclusion. Thus, the relevance of incentives “immediate price discount” and “free(or discounted) class upgrade” is predetermined by the used transport mode, country of residence and travel preferences.

“Price discount on future purchases” was the third highest-rated incentive. The regression analysis identified only one explanatory variable, “transport mode (carpooling/ride-sharing/shared taxi)” positively affecting interest in this incentive. The interest in this incentive drops with the increasing age and trip distance. Therefore, this type of incentive can be recommended especially to young people travelling by shared transport.

People with a master’s degree with respect to people without a university degree and those whose travel preference is meal inclusion and refundability are more likely to choose the incentive “being offered additional services”. The analysis indicate also higher interest in this incentive by people considering a low number of interchanges as a comfort solution. Four other explanatory variables with negative influence were identified as significant, two of which were travel preferences. Therefore, considering also positive regression coefficients, we can conclude that the relevance of this incentive can be to some extend revealed based on travellers’ preferences.

The following incentives were of moderate interest to respondents, as evidenced by the achieved average values lower than the value 3. The “Loyalty program with points collection to unlock different rewards” would be interesting for people who travel by shared services or walk. Likely, people using ride-sharing/carpooling/shared taxis already have experience with the loyalty program of the providers of these services, and therefore, they selected it. Results also show that people from the Czechia and Slovakia and those who need to carry special bags are interested in this stimulus.

The “provision of information on the solution’s environmental impact” was the best rated among the non-financial incentives. The occupation has a significant impact on selecting this incentive. Employed people or students are more likely to choose it. Contrarily, people living in Czechia and Slovakia are less likely to select this incentive than people from other analysed countries.

People who walk, travel for leisure, consider as comfortable feeling of personal safety while travelling and their preference is meal inclusion would be willing to change their travel decision due to “information about the positive aspects of a solution”.

The incentive “discounts on complementary services” could be interesting for people travelling to work or school, who prefer to have a meal included during travel or appreciate the cleanliness of stations and vehicles. Model results for the incentive “challenge you to achieve a specific goal” indicate that based on it people who travelled by bicycle on their last trip, employed people and students are more likely to change their travel choice. The incentive “competition with friends” could be relevant only for people travelling with their partners.

## 5 Discussion and conclusions

The aim of this article was to identify travellers’ perceptions towards travel offer categories and incentives in the journey planning context and the factors influencing them. We proposed a catalogue of travel offer categories and incentives, respectively. To find out which of the catalogue items are preferred by travellers, we designed and conducted a survey primarily in RIDE2RAIL demonstration countries. In order to determine who and in what context is interested in proposed travel offer categories and incentives, we built a data model for every offer category and incentive. Based on the results of ordinal response regression models, we identified a variety of factors influencing the selection of individual travel offer categories and incentives. These factors include trip characteristics as the mode of transport, trip origin and distance, perceptions of the comfort, and socio-demographic characteristics.

In general, travel offer categories received high ratings. Among the top categories, we find “reliable” and “quick”, which means that people prefer to spend as little time travelling as possible and be on time. These two factors are often considered critical quality attributes in public transport [33, 62, 63] and also significantly influence choice of transport mode [64, 65]. All other categories received a similar ratings with an average situated around the value 3 out of 5 except for the categories “philanthropic” and “social”. These results are in line with the studies of Friman et al. [66], Olsson et al. [67] and Sarriera et al. [68] who found out, that the possibility of socialisation is not a factor based on which travellers would choose a specific travel solution. Regarding results of the philanthropic category, Verplanken et al. [69] state that individual interest strongly impacts travel mode choice, while pro-social motives always stay behind. This may be the reason why interest in this category is so low. Although several categories achieved similar values, they can be ranked based on this evaluation and prioritised in order to gain clarity when displaying travel offers to travellers.

Compared to the travel offer categories, the incentives achieved a lower rating. Among the most rated are mainly financial incentives. According to our results, the preferred ways how to get incentivised to amend travel decisions are “immediate discount” and “class upgrade”. These findings support the current trend in journey planners’ design to modify travellers’ decisions by using credit systems with the possibility to change credits for financial incentives [44, 45]. Although our results indicate that financial incentives are preferred by travellers more than non-financial ones, literature shows that they are not very sustainable and do not have a lasting impact on changing travel behaviour [36, 38]. It is therefore important to look for forms of incentives that would be interesting for travellers on the one hand and sustainable on the other. In our research of the non-financial incentives, the respondents were most interested in the “loyalty program” with points collection to unlock different rewards and “environmental information”. The least interesting incentive for travellers was “a competition with friends”.

By using ordinal regression, for every travel offer category and incentive, several factors were found significant. The most recurring significant factor in the case of travel offer categories was gender and, in the case of incentives, age. These socio-demographic characteristics still play a significant role in planning and travel behaviour [70, 71], and therefore, they should be considered as strong criteria for recommendations in journey planners. The results also show that travellers’ choice of travel offer categories and incentives depends on different factors. The great variety of other identified factors underlines the importance of focusing on the personalisation of recommendations for travellers.

To implement travel offer categories into a journey planner, we need to identify and quantify contributing factors. In a case, if there is more than one relevant factor, they can be prioritised and weighted [72] to obtain for each category a single numeric value. To put numeric values on the same footing and make them more easily comparable, data transformations, such as z-score or min-max normalisation [73, p.114], can be applied. Final numerical values of travel offer categories can be presented, using visually attractive labels, together with travel offers and be further used as features describing travel offers in various machine learning tasks to extend journey planning functionalities. Implementation of incentives is methodologically simpler. For example, it is sufficient to define conditions which, if satisfied, the traveller is entitled to receive given incentives from the transport operator. In practice, the significant challenge might be a technical one, residing in ensuring application programming interfaces between transport operators and journey planning services to support the exchange of required information.

It is worth to reiterate that the use of travel offers categories can be justified by journey planners that offer to travellers many transport connections. Typically, such journey planners cover a large geographical area and connect several means of transport (e.g. a Europe-wide journey planner). Offer categories can help to travellers to identify faster which traveller offers correspond to they needs or be used as features characterising travel offers in machine learning tasks.

The research conducted in this paper has several limitations. The sample of respondents consisted mainly of people from Slovakia and Czechia, aged 18 to 50, with a university degree. Therefore, the interpretation of research results should be perceived primarily from the perspective of this group of people. Another limitation is that incentives were studied without considering a broader context (e.g., travel purpose, travel frequency, travellers’ motivations, etc.). This could contribute to low ratings received by non-financial incentives, which could become more attractive for travellers if combined with some externalities. For example, “provisions of information that can increase traveller’s awareness on the environmental sustainability of a travel offer” can be more relevant for a traveller if their employer monitors the environmental impact of business trips. In addition, only a few features characterising the demographic profile of travellers and travel behaviour are entering the analysis. Consequently, *R*^2^ values of the categorical regression model are relatively low. Nevertheless, according to conducted statistical tests, the results are significant.

Although the research contains the mentioned limitations, we consider the results to be valuable and relevant inputs in the initial stages of the design of journey planners. An ideal use case for our results would be a multimodal journey planner enabling searching for travel offers on a large geographical scale (e.g., across country borders or even Europe-wide). In such a situation, we can expect that travellers would appreciate a guidance on which travel solution to choose. However, such journey planners are currently rare, mainly due to difficulties with funding their development and operation. Perhaps, this could be resolved by an EU-wide policy prioritising support to the operation of such a journey planner or finding a way how to operate it via open collaboration like OpenStreetMap. For future work, we envisage further validation of the paper results by evaluating the level of satisfaction and usefulness of travel offer categories based on data collected during Ride2Rail demonstrations (i.e., the comparison of stated preferences with revealed preferences). It is also necessary to verify the ability of travel offer categories to be used as features in prediction models of travel offer choices made by travellers and identify traveller’s segments to enhance these prediction models.

## Supporting information

S1 File (PDF)
