# Supplementary material for: Study of travellers’ preferences towards travel offer categories and incentives in the journey planning context
Source: PLoS One. 2023 Apr 26;18(4):e0284844. doi: 10.1371/journal.pone.0284844 (PMC10132637; doi:10.1371/journal.pone.0284844)
Supplement: S1 File — (PDF) [file pone.0284844.s001.pdf]

A Appendix: Response variables

Variable name	Travel offer category name
OC[short]	Short
OC[cheap]	Cheap
OC[comf]	Comfort
OC[envir]	Environmentally friendly
OC[soc]	Social
OC[multi]	Multitasking
OC[door]	Door-to-door
OC[phil]	Philantropic
OC[quick]	Quick
OC[reliab]	Reliable
**Variable name**	**Incentive name**
IN[inf]	Additional information
IN[env.inf]	Environmental information
IN[goal]	Goal
IN[loyal]	Loyalty program
IN[fut.dis]	Future discount
IN[im.dis]	Immediate discount
IN[dis.ser]	Discount for services
IN[class.up]	Class upgrade
IN[ad.serv]	Additional services
IN[comp]	Competition

Table 3. Response variables.

B Appendix: Explanatory variables

Type	Variable name	Description
Trip related	REASN[business]<br>REASN[commute]<br>REASN[leisure]	The variables represent answers to Q1, which investigated reason of the chosen trip. Values "business", "commute" and "leisure", are represented by dummy variables, while the reference variable is the value "other".
	COMPN[colleague]<br>COMPN[family]<br>COMPN[partner]	The variables represent answers to Q2, which investigated with whom respondents travelled during the chosen trip. Values "colleague", "family", "partner", are represented by dummy variables, while the reference is value "alone".
	DISTAN	The variable represents answers to Q3, which investigated the length of the trip . It is an ordinal variable that acquires values: 1 (10km or less), 2 (10 to 50km), 3 (50-300km) or 4 (300km or more).
	ORIG[suburb]<br>ORIG[urban]	The variables represent answers to Q4, which investigated origin of the chosen trip. Values "urban area", "suburban area" are represented by dummy variables, while the reference variable is "rural area".

Type	Variable name	Description
	DEST[suburb]<br>DEST[urban]	The variables represent answers to Q5, which investigated where the chosen trip ended. Considered are values "urban area" and "suburban area", which are represented by dummy variables, while the reference value is "rural area".
	MODE[bike]<br>MODE[bus]<br>MODE[metro]<br>MODE[car]<br>MODE[share]<br>MODE[train]<br>MODE[walk]<br>MODE[other]	The variables represent answers to Q6, which investigated all transport means that were used during the chosen trips. Variables acquire value one if the respondents used the mean of transport in their trips and zero otherwise. Some variables include several transport means. The variable MODE[bike] includes bicycles and micro-mobility devices, the variable MODE[bus] includes also tram and trolleybus vehicles, the variable MODE[car] includes private cars, private taxi and motorbikes and the variable MODE[share] includes sharing services such as car pooling, ride sharing and shared taxi.
Preferences/Planning a trip	PREF[update]<br>PREF[meal]<br>PREF[connect]<br>PREF[refund]<br>PREF[seat]<br>PREF[company]<br>PREF[mode]<br>PREF[time]<br>PREF[interchange]<br>PREF[class]	The variables represent answers to Q7, which investigated respondents' travel preferences. Variables acquire value one if the respondents chose a corresponding travel preference and zero otherwise.
	NEED[animal]<br>NEED[infant]<br>NEED[bags]<br>NEED[health]<br>NEED[specbag]<br>NEED[noaddit]<br>NEED[other]	The variables represent answers to Q8, which investigated respondents' needs related to travelling. Variables acquire value one if the respondents chose the corresponding need and zero otherwise.
	COMF[clean]<br>COMF[seat]<br>COMF[no.modes]<br>COMF[no.interch]<br>COMF[privacy]<br>COMF[safety]<br>COMF[weath.prot]	The variables represent answers to Q11, which investigated what respondents consider as a comfortable solution. Variables acquire value one if the respondents chose the corresponding comfortable solution and zero otherwise.
Socio-demographic characteristics	GENDER[male]	The variable represents answers to Q15, which investigated gender of the respondents. Due to the unbalanced number of respondents' answers (see Figure 1A), we decided to encode the answers as two groups. Variable acquires value one if the respondent is male and zero otherwise.
	AGE	The variable represents answers to Q14 which investigated age of respondents. It is an ordinal variable that acquires values: 1 (less than 18), 2 (18-24), 3 (25-34), 4 (35-50), 5 (51-65) or 6 (more than 65).

Type	Variable name	Description
	COUNTRY[sk.cz]	The variable represents answers to Q16, which investigated the country in which respondents live. Due to the unbalanced number of respondents' answers (see Figure 1B), we decided to encode the answers as two groups. We merged respondents from Czechia and Slovakia to form one group due to their similar social, cultural and economic background. Thus, variable acquires value one if a respondent lives in Czechia or Slovakia and zero otherwise.
	EDU[bach]<br>EDU[mast]	The variables represent answers to Q17, which investigated the respondents level of education. Values "bachelor degree", "master's degree" are represented by dummy variables, while the reference value is "without university degree".
	OCCUP[stud]<br>OCCUP[empl]	The variable represent answers to Q18, which investigated respondents employment status. Values "employed" and "student" are represented by dummy variables, while the reference value is "other".

Table 4. Explanatory variables.

C Appendix: Questionnaire design

No.	Question	Types of answers
Q1	What was the reason of the trip you chose?	Single choice question:<br>Leisure, Business, Commute, Other commitments
Q2	Whom where you travelling with?	Single choice question:<br>Partner, Family, Friends, Other
Q3	How long was your trip?	Single choice question:<br>Very short (10 km or less), Short (10 to 50 km), Medium (50 to 300km), Long distance (300km or more)
Q4	Where did you start the trip from?	Single choice question:<br>An urban area (densely inhabited city), A suburban area (commuting zone), A rural area
Q5	What was your destination?	Single choice question:<br>An urban area (densely inhabited city), A suburban area (commuting zone), A rural area
Q6	Select all the means of transport you have used in that trip.	Multiple choice question:<br>On foot, Bicycle or micromibility, Metro, Bus/Tram/Trolleybus, Private car/Private taxi/Motorbike, Car pooling/Ride sharing/Shared taxi, Train, Plane, Ferry

No.	Question	Types of answers
Q7	What are the travel preferences you'd like to specify?	Multiple choice question: Specific means of transport, Specific transportation companies, A time interval for the departure and arrival times, A limit on the amount of transport changes, The travel class, The seat type, Meal inclusion, Refundability, Live notifications on trip status updates, On board connectivity
Q8	Do you have any of those needs?	Multiple choice question: I have large/multiple baggage, I have special baggage, I need animal allowance, I need help because of reduced mobility, No additional needs, I have health-related needs, I travel with infant, Other needs
Q9	Imagine that the travel app divides the available travel solutions into predefined categories. How much are you interested in travel solutions that are: quick, short, reliable, cheap, door-to-door, social, multitasking, environmentally-friendly, philanthropic, comfortable?	5-point scale: 1 - Not at all interested , 5 - Very interested
Q10	Now, if you could only choose some of these categories to make your final decision, which ones would you pick?	Multiple choice question (max 3 categories): Quick, Short, Reliable, Cheap, Door-to-door, Social, Multitasking, Environmentally friendly, Philanthropic, Comfortable
Q11	Which of these factors, in your opinion, define a comfortable solution?	Multiple choice question: Having a comfortable seat, Cleanliness of stations and vehicles, Low number of different means of transport, Minimum number of interchanges, High level of privacy, Feeling of personal safety, Protection from weather

No.	Question	Types of answers
Q12	Imagine you are inclined towards a choice and the app would like you to select a different travel solution. How likely would these incentives (immediate price discount, price discount on future purchases, loyalty program with points collection to unlock different rewards,being offered additional services, discounts on complementary services, free (or discounted) class upgrade, provide more information about the positive aspects of a solution, provide information on the solution's environmental impact, challenge you to achieve a specific goal, a competition with friends and a shared leaderboard with points assigned based on your travel choices) succeed?	5-point scale:<br>1 - Really unlikely, 5 - Totally likely
Q13	Can you think about anything else that could influence your final choice?	Open-ended question
Q14	How old are you?	Single choice question:<br>Less than 18, 18-24, 25-34, 35-50, 51-65, More than 65
Q15	What's your gender?	Single choice question:<br>Male, Female, Other, Prefer not to say
Q16	In which country do you live?	Open question
Q17	What is the highest degree or level of education you have completed?	Single choice question:<br>Basic education, Higher education, Bachelor's degree, Master's degree or higher, Prefer not to say
Q18	What is your current employment status?	Single choice question:<br>Employed full time, Employed part time, Unemployed and looking for job, Unemployed and not looking for job, Student, Retired, Self-employed, Unable to work, Prefer not to say

Table 5. Formulation of questions and types of answers.

D Appendix: Results of ordinal regression analysis - Travel offer categories

Predictor	OC[short]			OC[cheap]			OC[comf]			OC[envir]		
	β	p-value	exp(β)	β	p-value	exp(β)	β	p-value	exp(β)	β	p-value	exp(β)
REASN[business]	-0.1779	0.6438	0.8370	0.9585	0.0159	2.6078	0.5345	0.1787	1.7066	-0.1177	0.7580	0.8890
REASN[commute	0 .0597	0.8897	1.0615	0.6383	0.1402	1.8933	0.6562	0.1359	1.9275	-0.2642	0.5301	0.7678
REASN[leisure]	-0.0034	0.9928	0.9966	0.5416	0.1523	1.7188	0.6381	0.0981	1.8929	0.0121	0.9738	1.0122
COMPN[colleague	0.9855	0.0233	2.6792	-0.1691	0.6849	0.8444	-0.2359	0.5858	0.7899	-0.0503	0.9043	0.9509
COMPN[family]	0.1902	0.4512	1.2095	0.1908	0.4595	1.2102	-0.1412	0.5771	0.8683	0.1162	0.6481	1.1232
COMPN[partner]	0.1032	0.7318	1.1087	-0.0706	0.8226	0.9318	0.0437	0.8889	1.0447	0.4306	0.1449	1.5382
DISTAN	-0.5031	0.0000	0.6047	-0.1546	0.2050	0.8568	-0.1134	0.3583	0.8928	-0.1440	0.1192	0.8659
ORIG[suburb]	-0.6357	0.0184	0.5296	-0.0966	0.7250	0.9079	-0.1230	0.6560	0.8843	-0.2969	0.2660	0.7431
ORIG[urban]	-0.3669	0.0975	0.6929	-0.1067	0.6421	0.8988	-0.0915	0.6895	0.9126	-0.2870	0.2194	0.7505
DEST[suburb]	0.1722	0.6141	1.1879	0.6101	0.0763	1.8406	0.1662	0.6289	1.1808	0.1542	0.3384	1.1667
DEST[urban]	-0.0212	0.9368	0.9790	0.5103	0.0640	1.6658	0.4903	0.0771	1.6328	0.2190	0.2700	1.2448
MODE[walk]	-0.0268	0.8924	0.9736	0.1136	0.5790	1.1203	-0.1267	0.5366	0.8810	0.4403	0.0074	1.5532
MODE[bike]	0.1603	0.6286	1.1739	-0.2245	0.5046	0.7989	-1.1326	0.0008	0.3222	0.1555	0.3416	1.1682
MODE[metro]	0.7969	0.0437	2.2187	-0.1233	0.7821	0.8840	0.5181	0.1992	1.6788	-0.5613	0.3903	0.5705
MODE[bus]	0.1625	0.4834	1.1764	-0.2068	0.3912	0.8132	-0.2299	0.3399	0.7946	0.3385	0.2311	1.4028
MODE[car]	0.1863	0.4301	1.2048	-0.3015	0.2172	0.7397	0.3146	0.1977	1.3697	-0.1582	0.2363	0.8537
MODE[share]	0.4313	0.4099	1.5393	0.9480	0.0946	2.5805	1.4951	0.0144	4.4598	0.4918	0.5569	1.6353
MODE[train]	-0.0435	0.8428	0.9574	0.2504	0.2754	1.2845	-0.018	0.9371	0.9822	0.2893	0.2240	1.3355
MODE[other]	0.4510	0.4807	1.5699	1.1992	0.0716	3.3175	-0.4188	0.5206	0.6578	0.9986	0.6167	2.7145
PREF[mode]	0.1554	0.4327	1.1681	0.3222	0.1240	1.3802	-0.0729	0.7218	0.9297	0.1588	0.2047	1.1721
PREF[company]	0.1263	0.5935	1.1346	0.0147	0.9513	1.0148	-0.2013	0.4065	0.8177	-0.1646	0.2349	0.8482
PREF[time]	-0.2149	0.3648	0.8066	0.0226	0.9269	1.0229	-0.2562	0.2779	0.7740	-0.2217	0.2326	0.8012
PREF[interchange]	0.0344	0.8553	1.0350	-0.1639	0.4058	0.8488	-0.0542	0.7810	0.9472	-0.2233	0.1868	0.7999
PREF[class]	0.0515	0.8483	1.0528	0.3001	0.2998	1.3500	0.4407	0.1322	1.5538	-0.0917	0.2806	0.9124
PREF[seat]	0.1455	0.5299	1.1566	-0.1255	0.5959	0.8821	0.4132	0.0910	1.5116	-0.1753	0.2352	0.8392
PREF[meal]	-0.1079	0.7644	0.8977	0.9656	0.0098	2.6264	0.1969	0.5937	1.2176	0.0071	0.3529	1.0071
PREF[refund]	0.2020	0.3928	1.2238	0.5616	0.0254	1.7535	-0.2592	0.2857	0.7717	0.1082	0.2373	1.1143
PREF[update]	-0.4256	0.0243	0.6534	-0.1611	0.4071	0.8512	-0.1161	0.5479	0.8904	-0.1078	0.1895	0.8978
PREF[connect]	-0.0904	0.6701	0.9136	-0.1338	0.5396	0.8748	0.0601	0.7802	1.0619	-0.0141	0.2108	0.9860
NEED[bags]	-0.452	0.1163	0.6364	0.5132	0.0834	1.6706	0.2360	0.5755	1.2662	-0.2432	0.2930	0.7841
NEED[specbag]	-0.3879	0.2470	0.6785	-0.1766	0.5985	0.8381	0.1743	0.6017	1.1904	0.1494	0.3331	1.1611
NEED[animal]	0.1627	0.6915	1.1767	-0.1139	0.7780	0.8923	0.2360	0.5755	1.2662	0.1462	0.4109	1.1574
NEED[health]	-0.4418	0.2638	0.6429	0.1737	0.6969	1.1897	0.4854	0.3297	1.6248	-0.3510	0.4323	0.7040
NEED[infant]	0.3011	0.3929	1.3513	-0.2362	0.5044	0.7896	-0.3677	0.3190	0.6923	-0.0520	0.3606	0.9493
NEED[other]	-0.0126	0.9763	0.9875	-0.4252	0.3399	0.6536	0.0052	0.9909	1.0052	-0.0542	0.4410	0.9472
NEED[noaddit]	-0.5907	0.0553	0.5539	-0.0231	0.9423	0.9772	-0.4715	0.1393	0.6241	-0.3927	0.3172	0.6752
COMF[seat]	-0.1565	0.4432	0.8551	0.1636	0.4356	1.1777	0.6814	0.0012	1.9766	-0.5530	0.0071	0.5752
COMF[clean]	0.0484	0.8459	1.0496	0.2293	0.3786	1.2577	0.2225	0.3967	1.2492	0.4925	0.0499	1.6364
COMF[no.modes]	0.1080	0.5931	1.1140	0.0403	0.8496	1.0411	-0.1957	0.3613	0.8223	0.0433	0.2047	1.0443
COMF[no.interch	0.3395	0.0935	1.4042	0.1099	0.5985	1.1162	0.5951	0.0041	1.8132	0.0216	0.2035	1.0218
COMF[privacy]	-0.2091	0.3294	0.8113	-0.5471	0.0143	0.5786	0.3728	0.0925	1.4518	-0.1940	0.2132	0.8237
COMF[safety]	-0.0667	0.7384	0.9355	-0.1184	0.5686	0.8883	0.4186	0.0415	1.5198	0.4892	0.0146	1.6310
COMF[weath.prot]	0.3157	0.0858	1.3712	0.4281	0.0258	1.5343	-0.0454	0.8124	0.9556	-0.0037	0.1882	0.9963
GENDER[male]	-0.6037	0.0017	0.5468	-0.3937	0.0441	0.6746	-0.5288	0.0068	0.5893	-0.5509	0.0046	0.5764
AGE	-0.1369	0.2467	0.8721	-0.1781	0.1489	0.8369	-0.2025	0.0985	0.8167	0.1228	0.1209	1.1307
COUNTRY[sk.cz]	0.1358	0.5523	1.1455	-0.9093	0.0001	0.4028	0.3512	0.1318	1.4208	-0.5216	0.0223	0.5936
EDU[bach]	-0.2970	0.2544	0.7430	0.0613	0.8217	1.0632	0.0955	0.7173	1.1002	0.2108	0.2657	1.2347
EDU[mast]	-0.1375	0.6015	0.8715	-0.0436	0.8726	0.9573	0.2562	0.3249	1.2920	-0.2554	0.2660	0.7746
OCCUP[empl]	-0.0347	0.9359	0.9659	-0.4947	0.2845	0.6098	-1.0796	0.0263	0.3397	0.4040	0.4632	1.4978
OCCUP[stud]	0.2022	0.6293	1.2241	-0.0724	0.8807	0.9302	-1.1298	0.0173	0.3231	0.1411	0.4478	1.1515

Table 6. Results for short, cheap, comfortable and environmentally friendly travel offer categories. Shaded cells indicate statistically significant predictors.

Predictor	OC[soc]			OC[multi]			OC[door]			OC[phil]		
	β	p-value	exp(β)	β	p-value	exp(β)	β	p-value	exp(β)	β	p-value	exp(β)
REASN[business]	-0.1108	0.7856	0.8951	0.1134	0.7634	1.1201	-0.1366	0.7239	0.8723	-0.5008	0.2016	0.6060
REASN[commute]	0.1333	0.7683	1.1426	0.3290	0.4279	1.3896	-0.2298	0.5866	0.7947	-0.1374	0.7501	0.8716
REASN[leisure]	0.0097	0.9803	1.0097	0.0966	0.7917	1.1014	0.2323	0.5323	1.2615	-0.4292	0.2531	0.6510
COMPN[colleague]	0.2416	0.5729	1.2733	0.1791	0.6567	1.1961	0.0022	0.9956	1.0022	0.3538	0.3933	1.4245
COMPN[family]	-0.1078	0.6762	0.8978	0.1740	0.4692	1.1901	-0.3408	0.1783	0.7112	0.1383	0.5829	1.1483

Predictor	OC[soc]			OC[multi]			OC[door]			OC[phil]		
	β	p-value	exp(β)	β	p-value	exp(β)	β	p-value	exp(β)	β	p-value	exp(β)
COMPN[partner]	-0.3045	0.3302	0.7375	0.1900	0.5218	1.2092	-0.6511	0.0400	0.5215	-0.1552	0.6236	0.8562
DISTAN	-0.056	0.6509	0.9455	-0.0595	0.6129	0.9422	0.0023	0.9848	1.0023	-0.1538	0.2070	0.8574
ORIG[suburb]	-0.6536	0.0181	0.5202	-0.5731	0.0295	0.5638	-0.3736	0.1652	0.6883	-0.1709	0.5332	0.8429
ORIG[urban]	-0.6506	0.0044	0.5217	-0.5961	0.0074	0.5510	-0.2268	0.3019	0.7971	0.0540	0.8147	1.0555
DEST[suburb]	0.6552	0.0661	1.9255	0.0984	0.7674	1.1034	0.3420	0.3233	1.4078	-0.4128	0.2505	0.6618
DEST[urban]	0.1221	0.6597	1.1299	-0.0142	0.9573	0.9859	-0.1259	0.6449	0.8817	0.0947	0.7362	1.0993
MODE[walk]	0.1686	0.4203	1.1836	0.2572	0.1881	1.2933	-0.2333	0.2407	0.7919	-0.2219	0.2769	0.801
MODE[bike]	-0.0437	0.9027	0.9572	0.1901	0.5716	1.2094	0.1268	0.7057	1.1352	-0.2752	0.4513	0.7594
MODE[metro]	0.3181	0.4475	1.3745	0.5892	0.1306	1.8025	0.3998	0.3008	1.4915	0.4669	0.2490	1.595
MODE[bus]	-0.1060	0.6717	0.8994	-0.0824	0.7153	0.9209	-0.2791	0.2273	0.7565	0.6989	0.0043	2.0115
MODE[car]	0.4289	0.0854	1.5356	0.0718	0.7539	1.0744	0.3245	0.1631	1.3833	0.5954	0.0155	1.8138
MODE[share]	1.7430	0.0018	5.7145	1.3445	0.0090	3.8363	0.3362	0.5364	1.3996	1.1073	0.0398	3.0262
MODE[train]	-0.1099	0.6326	0.8959	0.5294	0.0147	1.6979	-0.4403	0.0492	0.6438	0.0980	0.6706	1.1030
MODE[other]	0.2012	0.7278	1.2229	-0.2371	0.6906	0.7889	0.3850	0.5540	1.4696	0.3586	0.5675	1.4313
PREF[mode]	0.1433	0.5042	1.1541	0.0278	0.8881	1.0282	0.3311	0.3670	1.3925	0.4766	0.0224	1.6106
PREF[company]	-0.3800	0.1367	0.6839	0.0548	0.8137	1.0563	-0.0338	0.8871	0.9668	-0.6136	0.0156	0.5414
PREF[time]	-0.2454	0.3118	0.7824	-0.2363	0.3089	0.7895	-0.1898	0.4256	0.8271	-0.1971	0.4092	0.8211
PREF[interchange	-0.4185	0.0367	0.6580	-0.3192	0.0882	0.7267	-0.2259	0.2320	0.7978	-0.0458	0.8164	0.9552
PREF[class]	-0.1281	0.6666	0.8798	0.0487	0.8572	1.0499	0.2707	0.3366	1.3109	-0.4408	0.1345	0.6435
PREF[seat]	0.0881	0.7243	1.0921	0.4978	0.0326	1.6451	0.0362	0.8773	1.0369	0.4085	0.0958	1.5046
PREF[meal]	0.9222	0.0104	2.5148	0.7562	0.0321	2.1302	0.3311	0.3670	1.3925	0.5925	0.0976	1.8085
PREF[refund]	0.2874	0.2455	1.3330	0.1012	0.6636	1.1065	-0.1721	0.4797	0.8419	0.0160	0.9473	1.0161
PREF[update]	-0.0521	0.7944	0.9492	0.0115	0.9503	1.0116	-0.0221	0.9067	0.9781	0.1795	0.3605	1.1966
PREF[connect]	-0.1489	0.5012	0.8617	0.3621	0.0766	1.4363	-0.2899	0.1656	0.7483	-0.0393	0.8545	0.9615
NEED[bags]	-0.2315	0.4434	0.7933	-0.1171	0.6769	0.8895	0.6627	0.0250	1.9400	-0.8288	0.0046	0.4366
NEED[specbag]	0.0853	0.8031	1.0890	0.4157	0.2207	1.5154	0.4558	0.1904	1.5774	0.3323	0.3310	1.3942
NEED[animal]	0.5520	0.1561	1.7367	0.0497	0.8963	1.0510	0.6975	0.0850	2.0087	-0.9422	0.0200	0.3898
NEED[health]	0.8304	0.0575	2.2942	-0.375	0.3831	0.6873	0.4347	0.2806	1.5445	0.5521	0.2349	1.7369
NEED[infant]	0.0335	0.9258	1.0341	0.6033	0.0698	1.8281	0.3649	0.3107	1.4404	0.0877	0.8063	1.0917
NEED[other]	-0.1063	0.8143	0.8992	-0.1593	0.7178	0.8527	0.1223	0.7894	1.1301	-0.5208	0.2532	0.594
NEED[noaddit]	-0.0762	0.8171	0.9266	-0.0122	0.9680	0.9879	0.9889	0.0021	2.6883	-0.8159	0.0106	0.4422
COMF[seat]	-0.1843	0.3774	0.8317	0.0844	0.6669	1.0881	0.0644	0.7542	1.0665	-0.1077	0.6028	0.8979
COMF[clean]	0.4981	0.0801	1.6456	0.2673	0.2839	1.3064	0.4431	0.0875	1.5575	0.1076	0.6872	1.1136
COMF[no.modes]	-0.292	0.1768	0.7468	0.0928	0.6397	1.0972	0.3099	0.1297	1.3633	0.0056	0.9785	1.0056
COMF[no.interch]	-0.1832	0.3895	0.8326	-0.0927	0.6404	0.9115	0.6329	0.0021	1.8831	-0.0780	0.7138	0.9250
COMF[privacy]	-0.7532	0.0016	0.4709	-0.1473	0.4798	0.8630	0.0280	0.8961	1.0284	-0.1934	0.3833	0.8242
COMF[safety]	0.5575	0.0099	1.7463	0.1230	0.5273	1.1309	0.3377	0.0921	1.4017	0.4413	0.0327	1.5547
COMF[weath.prot]	-0.1131	0.5669	0.8931	0.0632	0.7278	1.0652	0.0520	0.7822	1.0534	-0.0799	0.6807	0.9232
GENDER[male]	-0.2838	0.1592	0.7529	-0.1982	0.2888	0.8202	-0.3810	0.0454	0.6832	-1.0131	0.0000	0.3631
AGE	-0.1173	0.3503	0.8893	-0.1348	0.2562	0.8739	0.1453	0.2361	1.1564	-0.1467	0.2427	0.8636
COUNTRY[sk.cz]	-0.0549	0.8197	0.9466	-0.1825	0.4179	0.8332	-0.2409	0.2922	0.7859	-0.6113	0.0099	0.5426
EDU[bach]	-0.4140	0.1228	0.6610	0.2269	0.3750	1.2547	0.5563	0.0311	1.7442	-0.1257	0.6401	0.8819
EDU[mast]	-0.4418	0.1006	0.6429	0.2702	0.2923	1.3102	0.6939	0.0074	2.0015	-0.4195	0.1177	0.6574
OCCUP[empl]	0.8098	0.0925	2.2475	0.0993	0.8222	1.1044	0.4776	0.2812	1.6122	1.0063	0.0397	2.7355
OCCUP[stud]	0.9126	0.0637	2.4908	-0.0009	0.9985	0.9991	0.5722	0.2078	1.7722	0.7245	0.1435	2.0637

Table 7. Results for social, multitasking, door-to-door and philanthropic travel offer categories. Shaded cells indicate statistically significant predictors.

E Appendix: Results of ordinal regression analysis - Incentive categories

Predictor	IN[im.dis]			IN[fut.dis]			IN[loyal]			IN[ad.serv]		
	β	p-value	exp(β)	β	p-value	exp(β)	β	p-value	exp(β)	β	p-value	exp(β)
REASN[business]	0.1884	0.6266	1.2073	0.2696	0.4500	1.3094	0.1393	0.7051	1.1495	0.0958	0.8095	1.1005
REASN[commute]	-0.0667	0.8747	0.9355	0.4111	0.3038	1.5085	0.3414	0.4058	1.4069	-0.4053	0.3525	0.6668
REASN[leisure]	0.1625	0.6706	1.1764	0.2224	0.5267	1.2491	0.3310	0.3541	1.3924	-0.0961	0.8041	0.9084
COMPN[colleague	0.2516	0.5621	1.2861	-0.5105	0.2276	0.6002	-0.2769	0.4987	0.7581	0.6656	0.1184	1.9457

Predictor	IN[im.dis]			IN[fut.dis]			IN[loyal]			IN[ad.serv]		
	β	p-value	exp(β)	β	p-value	exp(β)	β	p-value	exp(β)	β	p-value	exp(β)
COMPN[family]	0.1097	0.6726	1.1159	0.0282	0.9072	1.0286	0.0167	0.9447	1.0168	0.3852	0.1279	1.4699
COMPN[partner]	-0.1330	0.6614	0.8755	-0.1920	0.5257	0.8253	-0.0656	0.8212	0.9365	-0.0049	0.9872	0.9951
DISTAN	-0.1398	0.2623	0.8695	-0.2788	0.0212	0.7567	-0.2227	0.0588	0.8004	-0.1173	0.3240	0.8893
ORIG[suburb]	0.2085	0.4476	1.2318	-0.4918	0.0631	0.6115	-0.2472	0.3472	0.7810	0.0572	0.8310	1.0589
ORIG[urban]	0.0453	0.8441	1.0463	-0.3527	0.1073	0.7028	-0.0107	0.9608	0.9894	0.2185	0.3276	1.2442
DEST[suburb]	0.2479	0.4810	1.2813	0.3912	0.2505	1.4788	0.1446	0.6686	1.1556	-0.3969	0.2634	0.6724
DEST[urban]	0.2234	0.4204	1.2503	0.3971	0.2671	1.4875	0.2237	0.3887	1.2507	-0.3350	0.2151	0.7153
MODE[walk]	-0.0993	0.6241	0.9055	0.2189	0.3454	1.2447	0.4431	0.0222	1.5575	0.0976	0.6237	1.1025
MODE[bike]	0.0399	0.9067	1.0407	-0.0236	0.9435	0.9767	-0.4671	0.1575	0.6268	-0.3325	0.3206	0.7171
MODE[metro]	-0.1643	0.6896	0.8485	-0.3586	0.3791	0.6987	-0.3485	0.3838	0.7057	0.6577	0.0941	1.9303
MODE[bus]	0.5563	0.0222	1.7442	0.2189	0.2320	1.2447	-0.1445	0.5265	0.8655	0.3441	0.1365	1.4107
MODE[car]	0.1003	0.6783	1.1055	0.2307	0.3252	1.2595	-0.1657	0.4707	0.8473	0.1636	0.4822	1.1777
MODE[share]	0.1222	0.8237	1.1300	1.1707	0.0328	3.2242	1.1329	0.0251	3.1046	0.6486	0.2392	1.9129
MODE[train]	0.0508	0.8252	1.0521	-0.1391	0.5317	0.8701	-0.1695	0.4470	0.8441	-0.3685	0.0939	0.6918
MODE[other]	0.6886	0.2712	1.9909	-0.3710	0.5335	0.6900	-0.0098	0.9868	0.9902	0.4001	0.5172	1.4920
PREF[mode]	0.0198	0.9238	1.0200	0.4452	0.2120	1.5608	-0.0302	0.8770	0.9703	-0.1001	0.6174	0.9047
PREF[company]	0.3439	0.1499	1.4104	0.2385	0.2484	1.2693	-0.2912	0.2142	0.7474	-0.0813	0.7322	0.9219
PREF[time]	0.1146	0.6361	1.1214	-0.3372	0.1472	0.7138	0.2472	0.2816	1.2804	-0.8130	0.0007	0.4435
PREF[interchange]	0.0981	0.6112	1.1031	0.0484	0.8364	1.0496	-0.0759	0.6841	0.9269	-0.5024	0.0086	0.6051
PREF[class]	0.3432	0.2181	1.4095	0.1829	0.4278	1.2007	-0.0086	0.9746	0.9914	0.1776	0.5203	1.1943
PREF[seat]	0.0638	0.7845	1.0659	-0.2896	0.1430	0.7486	0.1766	0.4392	1.1932	0.1024	0.6622	1.1078
PREF[meal]	0.2452	0.5004	1.2779	0.3203	0.1783	1.3775	-0.1248	0.7151	0.8827	1.3237	0.0003	3.7573
PREF[refund]	0.2618	0.2833	1.2993	-0.0738	0.6947	0.9289	0.1414	0.5401	1.1519	0.5289	0.0273	1.6971
PREF[update]	-0.1788	0.3632	0.8363	0.3070	0.2602	1.3593	-0.0934	0.6172	0.9108	-0.0288	0.8809	0.9716
PREF[connect]	0.4934	0.0237	1.6379	0.6022	0.1203	1.8261	0.1153	0.5776	1.1222	0.2107	0.3152	1.2345
NEED[bags]	-0.2998	0.3062	0.7410	-0.1247	0.7092	0.8828	-0.1900	0.4958	0.8270	0.2801	0.3292	1.3233
NEED[specbag]	0.0637	0.8551	1.0658	-0.1333	0.6450	0.8752	0.7603	0.0198	2.1389	0.5160	0.1344	1.6753
NEED[animal]	0.2587	0.5292	1.2952	-0.1580	0.6055	0.8538	-0.5558	0.1459	0.5736	0.1362	0.7330	1.1459
NEED[health]	-0.6827	0.1100	0.5053	-0.5660	0.1931	0.5678	0.4044	0.3479	1.4984	0.0730	0.8721	1.0757
NEED[infant]	-0.0274	0.9383	0.9730	0.1174	0.7757	1.1246	-0.4499	0.1865	0.6377	0.0725	0.8343	1.0752
NEED[other]	-0.0309	0.9447	0.9696	0.1798	0.5911	1.1970	-0.2477	0.5715	0.7806	0.2102	0.6375	1.2339
NEED[noaddit]	-0.3602	0.2594	0.6975	0.1013	0.6950	1.1066	-0.1666	0.5891	0.8465	0.0596	0.8490	1.0614
COMF[seat]	-0.0524	0.8018	0.9489	-0.0588	0.7687	0.9429	-0.1453	0.4636	0.8648	0.2878	0.1580	1.3335
COMF[clean]	0.1169	0.6609	1.1240	-0.1148	0.5665	0.8915	0.0989	0.6932	1.1040	0.4421	0.0869	1.5560
COMF[no.modes]	-0.3249	0.1229	0.7226	-0.0405	0.8414	0.9603	-0.4122	0.0362	0.6622	-0.4053	0.0472	0.6668
COMF[no.interch]	0.3125	0.1331	1.3668	0.0191	0.9292	1.0193	-0.0188	0.9252	0.9814	0.4631	0.0256	1.5890
COMF[privacy]	-0.3013	0.1665	0.7399	-0.045	0.8223	0.9560	-0.0670	0.7471	0.9352	0.1751	0.4179	1.1914
COMF[safety]	0.0252	0.9029	1.0255	-0.3205	0.0844	0.7258	0.2243	0.2557	1.2514	0.3595	0.0732	1.4326
COMF[weath.prot]	0.1633	0.3932	1.1774	-0.2788	0.0212	0.7567	0.0598	0.7441	1.0616	-0.0067	0.9719	0.9933
GENDER[male]	-0.1586	0.4203	0.8533	0.1947	0.3076	1.2149	-0.3119	0.0969	0.7321	-0.0777	0.6845	0.9252
AGE	-0.2593	0.0292	0.7716	-0.2374	0.0387	0.7887	-0.2275	0.0502	0.7965	-0.2435	0.0392	0.7839
COUNTRY[sk.cz]	-0.9943	0.0000	0.3700	-0.2652	0.2419	0.7671	0.5023	0.0257	1.6525	0.3340	0.1525	1.3965
EDU[bach]	-0.1845	0.4914	0.8315	-0.1160	0.6567	0.8905	-0.0590	0.8190	0.9427	0.3547	0.1814	1.4258
EDU[mast]	0.0682	0.7986	1.0706	-0.1713	0.5075	0.8426	0.0558	0.8308	1.0574	0.7933	0.0028	2.2107
OCCUP[empl]	0.0919	0.8338	1.0963	-0.6910	0.0984	0.5011	-0.2529	0.5542	0.7765	-0.0268	0.9525	0.9736
OCCUP[stud]	0.5566	0.2310	1.7447	-0.7230	0.0993	0.4853	-0.4727	0.2914	0.6233	0.2065	0.6602	1.2294

Table 8. Results for the incentives: Immediate price discount, Price discount on future purchases, Loyalty program, Additional services. Shaded cells indicate statistically significant predictors.

Predictor	IN[dis.ser]			IN[class.up]			IN[inf]			IN[env.inf]		
	β	p-value	exp(β)	β	p-value	exp(β)	β	p-value	exp(β)	β	p-value	exp(β)
REASN[business]	0.7132	0.0563	2.0405	0.3529	0.3663	1.4232	0.5657	0.1454	1.7607	-0.2891	0.4656	0.7489
REASN[commute]	0.8844	0.0319	2.4215	0.3135	0.4686	1.3682	0.3252	0.4426	1.3843	-0.4288	0.3152	0.6513
REASN[leisure]	0.6700	0.0620	1.9542	-0.1892	0.6213	0.8276	0.8196	0.0299	2.2696	-0.2233	0.5592	0.7999
COMPN[colleague]	-0.1586	0.6969	0.8533	-0.3428	0.3953	0.7098	0.1285	0.7497	1.1371	0.0362	0.9273	1.0369
COMPN[family]	-0.1176	0.6319	0.8891	0.3449	0.1761	1.4118	0.1951	0.4308	1.2154	0.4532	0.0689	1.5733
COMPN[partner]	0.2223	0.4633	1.2489	0.5284	0.0894	1.6962	-0.0093	0.9752	0.9907	0.3697	0.2203	1.4473
DISTAN	-0.1516	0.2011	0.8593	-0.0399	0.7421	0.9609	0.0524	0.6547	1.0538	-0.0569	0.6294	0.9447
ORIG[suburb]	-0.0728	0.7827	0.9298	-0.0494	0.8549	0.9518	-0.6779	0.0120	0.5077	-0.5203	0.0509	0.5943
ORIG[urban]	0.0958	0.6665	1.1005	0.1895	0.4096	1.2086	-0.4204	0.0605	0.6568	-0.3978	0.0719	0.6718

Predictor	IN[dis.ser]			IN[class.up]			IN[inf]			IN[env.inf]		
	β	p-value	exp(β)	β	p-value	exp(β)	β	p-value	exp(β)	β	p-value	exp(β)
DEST[suburb]	-0.6707	0.0464	0.5114	-0.3859	0.2640	0.6798	0.2949	0.3885	1.3430	-0.1673	0.6225	0.8459
DEST[urban]	-0.1934	0.4554	0.8242	-0.1431	0.6025	0.8667	0.1454	0.5867	1.1565	-0.0599	0.8250	0.9419
MODE[walk]	0.1836	0.3468	1.2015	-0.3782	0.0620	0.6851	0.3941	0.0448	1.4830	0.2102	0.2819	1.2339
MODE[bike]	0.0286	0.9315	1.0290	-0.6446	0.0614	0.5249	0.0807	0.8021	1.0840	0.5929	0.0681	1.8092
MODE[metro]	-0.3431	0.3887	0.7096	0.2101	0.5952	1.2338	-0.1488	0.7056	0.8617	-0.0496	0.9006	0.9516
MODE[bus]	0.1841	0.4265	1.2021	0.5740	0.0171	1.7754	0.2476	0.2741	1.2809	0.3431	0.1333	1.4093
MODE[car]	0.2833	0.2311	1.3275	0.2445	0.3066	1.2770	0.1989	0.3817	1.2201	0.1938	0.3993	1.2139
MODE[share]	0.6133	0.2742	1.8465	0.3542	0.5052	1.4250	0.3471	0.5326	1.4150	0.1290	0.8066	1.1377
MODE[train]	-0.2839	0.1987	0.7528	-0.2933	0.1951	0.7458	-0.4105	0.0632	0.6633	0.1406	0.5216	1.1510
MODE[other]	-0.5164	0.3858	0.5967	-0.2267	0.7181	0.7972	0.2517	0.6587	1.2862	0.3203	0.5885	1.3775
PREF[mode]	-0.0182	0.9263	0.9820	0.0815	0.6901	1.0849	0.1932	0.3235	1.2131	-0.0049	0.9804	0.9951
PREF[company]	0.2278	0.3434	1.2558	0.0818	0.7393	1.0852	0.2232	0.3418	1.2501	-0.1625	0.4808	0.8500
PREF[time]	-0.0522	0.8184	0.9491	-0.3853	0.1030	0.6802	-0.3161	0.1764	0.7290	-0.1558	0.4901	0.8557
PREF[interchange]	-0.1128	0.5431	0.8933	-0.2838	0.1448	0.7529	-0.1878	0.3147	0.8288	-0.1301	0.4827	0.8780
PREF[class]	0.1160	0.6712	1.1230	0.4325	0.1273	1.5411	-0.3083	0.2646	0.7347	-0.2458	0.3728	0.7821
PREF[seat]	-0.2048	0.3782	0.8148	-0.4436	0.0600	0.6417	-0.2914	0.2070	0.7472	-0.0789	0.7294	0.9241
PREF[meal]	0.7825	0.0345	2.1869	0.8597	0.0182	2.3625	0.9404	0.0087	2.5610	0.5234	0.1370	1.6878
PREF[refund]	0.4336	0.0700	1.5428	0.1549	0.5177	1.1675	0.0453	0.8458	1.0463	0.1047	0.6545	1.1104
PREF[update]	0.1276	0.4911	1.1361	0.1254	0.5116	1.1336	-0.1240	0.5048	0.8834	-0.2784	0.1355	0.7570
PREF[connect]	0.0890	0.6641	1.0931	0.1998	0.3485	1.2212	0.3354	0.0986	1.3985	0.1254	0.5384	1.1336
NEED[bags]	0.0000	0.9999	1.0000	0.1190	0.6874	1.1264	-0.4429	0.1175	0.6422	-0.0790	0.7815	0.9240
NEED[specbag]	0.2325	0.4880	1.2618	0.3790	0.2759	1.4608	0.2563	0.4404	1.2921	0.2750	0.4007	1.3165
NEED[animal]	-0.3447	0.3659	0.7084	0.0275	0.9466	1.0279	-0.4529	0.2302	0.6358	-0.0968	0.8019	0.9077
NEED[health]	0.1184	0.7756	1.1257	0.1381	0.7523	1.1481	0.2961	0.4902	1.3446	-0.0364	0.9322	0.9643
NEED[infant]	-0.5204	0.1294	0.5943	-0.366	0.2992	0.6935	-0.0801	0.8102	0.9230	0.0465	0.8893	1.0476
NEED[other]	-0.0805	0.8561	0.9227	0.2115	0.6354	1.2355	-0.3197	0.4753	0.7264	0.2872	0.5268	1.3327
NEED[noaddit]	-0.5583	0.0715	0.5722	-0.0868	0.7852	0.9169	-0.5354	0.0806	0.5854	-0.3016	0.3307	0.7396
COMF[seat]	0.2968	0.1344	1.3455	0.7530	0.0003	2.1234	-0.1771	0.3808	0.8377	-0.5524	0.0068	0.5756
COMF[clean]	0.4983	0.0444	1.6459	0.4918	0.0693	1.6353	0.3480	0.1616	1.4162	0.0248	0.9219	1.0251
COMF[no.modes]	0.3281	0.1052	1.3883	-0.3531	0.0888	0.7025	-0.3626	0.0731	0.6959	-0.3609	0.0771	0.6970
COMF[no.interch]	0.1973	0.3310	1.2181	0.2452	0.2419	1.2779	0.0819	0.6818	1.0853	-0.1224	0.5425	0.8848
COMF[privacy]	-0.2198	0.2956	0.8027	0.3387	0.1247	1.4031	-0.0431	0.8413	0.9578	-0.2614	0.2179	0.7700
COMF[safety]	0.3846	0.0523	1.4690	0.0052	0.9795	1.0052	0.5166	0.0095	1.6763	0.3083	0.1199	1.3611
COMF[weath.prot	-0.3170	0.0855	0.7283	0.0073	0.9692	1.0073	-0.2811	0.1319	0.7550	-0.0107	0.9543	0.9894
GENDER[male]	-0.2478	0.1922	0.7805	-0.0844	0.6663	0.9191	-0.353	0.0594	0.7026	-0.5450	0.0041	0.5798
AGE	-0.0368	0.7493	0.9639	-0.2951	0.0137	0.7445	0.0537	0.6375	1.0552	0.1859	0.1129	1.2043
COUNTRY[sk.cz]	0.1291	0.5685	1.1378	0.5263	0.0260	1.6927	0.0492	0.8252	1.0504	-0.5418	0.0183	0.5817
EDU[bach]	0.2331	0.3694	1.2625	-0.0292	0.913	0.9712	0.1306	0.6197	1.1395	0.3890	0.1363	1.4755
EDU[mast]	0.0292	0.9110	1.0296	-0.0859	0.7467	0.9177	0.0927	0.7202	1.0971	-0.1568	0.5411	0.8549
OCCUP[empl]	-0.0705	0.8682	0.9319	-0.8400	0.0772	0.4317	0.0491	0.9077	1.0503	0.9271	0.0399	2.5272
OCCUP[stud]	-0.2230	0.615	0.8001	-0.8164	0.1012	0.442	0.1916	0.6654	1.2112	0.9539	0.0406	2.5958

Table 9. Results for the incentives: Discounts on complementary services, Free (or discounted) class upgrade, More information about positive aspects of a solution, Information on the solution's environmental impact. Shaded cells indicate statistically significant predictors.

Predictor	IN[goal]			IN[comp]		
	β	p-value	exp(β)	β	p-value	exp(β)
REASN[business]	0.5599	0.1389	1.7505	0.2079	0.5977	1.2311
REASN[commute]	0.4836	0.2423	1.6219	0.1260	0.7675	1.1343
REASN[leisure]	-0.0531	0.8846	0.9483	0.0858	0.8189	1.0896
COMPN[colleague]	-0.2533	0.5342	0.7762	-0.0200	0.9617	0.9802
COMPN[family]	0.2228	0.3712	1.2496	0.3563	0.1616	1.4280
COMPN[partner]	-0.1323	0.6639	0.8761	0.6980	0.0248	2.0097
DISTAN	0.0343	0.7773	1.0349	0.0128	0.9163	1.0129
ORIG[suburb]	-0.2947	0.2693	0.7448	-0.1411	0.5993	0.8684
ORIG[urban]	-0.6459	0.0041	0.5242	-0.1801	0.4192	0.8352
DEST[suburb]	-0.2681	0.4333	0.7648	0.2940	0.3979	1.3418
DEST[urban]	-0.1373	0.6083	0.8717	0.3785	0.1654	1.4601
MODE[walk]	0.1478	0.4576	1.1593	0.2068	0.3011	1.2297
MODE[bike]	0.8706	0.0111	2.3883	0.3985	0.2322	1.4896

Predictor	IN[goal]			IN[comp]		
	β	p-value	exp(β)	β	p-value	exp(β)
MODE[metro]	0.0700	0.8604	1.0725	0.0733	0.8556	1.0761
MODE[bus]	0.3113	0.1830	1.3652	0.0823	0.7230	1.0858
MODE[car]	0.4038	0.0922	1.4975	-0.0301	0.8987	0.9703
MODE[share]	1.0443	0.0754	2.8414	0.9781	0.0702	2.6594
MODE[train]	-0.4669	0.0390	0.6269	0.0586	0.7957	1.0604
MODE[other]	-0.5764	0.3384	0.5619	-0.9582	0.1226	0.3836
PREF[mode]	0.0262	0.8955	1.0265	-0.1560	0.4446	0.8556
PREF[company]	-0.3737	0.1093	0.6882	-0.4865	0.0434	0.6148
PREF[time]	-0.0544	0.8109	0.9471	-0.2048	0.3852	0.8148
PREF[interchange]	0.0801	0.6686	1.0834	-0.5296	0.0060	0.5888
PREF[class]	0.0363	0.8965	1.0370	-0.2910	0.3081	0.7475
PREF[seat]	-0.3959	0.0903	0.6731	-0.3584	0.1321	0.6988
PREF[meal]	0.5952	0.0956	1.8134	0.6247	0.0765	1.8677
PREF[refund]	0.1086	0.6507	1.1147	-0.1247	0.6022	0.8828
PREF[update]	-0.1508	0.4209	0.8600	-0.0131	0.9449	0.9870
PREF[connect]	0.3856	0.0680	1.4705	0.2071	0.3313	1.2301
NEED[bags]	0.0492	0.8636	1.0504	-0.5826	0.0522	0.5584
NEED[specbag]	0.6409	0.0582	1.8982	0.2275	0.5028	1.2555
NEED[animal]	0.1047	0.7845	1.1104	-0.3292	0.3987	0.7195
NEED[health]	0.2964	0.5242	1.3450	0.2434	0.5743	1.2756
NEED[infant]	0.3727	0.2820	1.4516	0.1577	0.6551	1.1708
NEED[other]	-0.6715	0.1502	0.5109	-0.6715	0.1370	0.5109
NEED[noaddit]	-0.1488	0.6327	0.8617	-0.5775	0.0771	0.5613
COMF[seat]	-0.0724	0.7205	0.9302	-0.1961	0.3355	0.8219
COMF[clean]	-0.0592	0.8143	0.9425	0.4541	0.0853	1.5748
COMF[no.modes]	-0.3570	0.0762	0.6998	-0.2042	0.3171	0.8153
COMF[no.interch]	-0.0137	0.9457	0.9864	0.1829	0.3746	1.2007
COMF[privacy]	-0.2939	0.1642	0.7454	-0.084	0.7028	0.9194
COMF[safety]	0.0395	0.8454	1.0403	0.2668	0.1873	1.3058
COMF[weath.prot]	-0.3493	0.0606	0.7052	0.1332	0.4848	1.1425
GENDER[male]	-0.5191	0.0063	0.5951	-0.3651	0.0607	0.6941
AGE	-0.0980	0.3989	0.9066	-0.3430	0.0038	0.7096
COUNTRY[sk.cz]	-0.0619	0.7832	0.9400	-0.1194	0.6017	0.8875
EDU[bach]	-0.0668	0.7985	0.9354	-0.2650	0.3125	0.7672
EDU[mast]	-0.0929	0.7186	0.9113	-0.3988	0.1371	0.6711
OCCUP[empl]	1.0212	0.0239	2.7765	0.2834	0.5315	1.3276
OCCUP[stud]	0.8724	0.0467	2.3926	-0.2223	0.6347	0.8007

Table 10. Results for the incentives: Challenge to achieve a specific goal,Competition with friends. Shaded cells indicate statistically significant predictors.
